# The Role of Metal Tolerance Proteins (MTPs) Associated with the Homeostasis of Divalent Mineral Elements in Ga-Treated Rice Plants

**DOI:** 10.3390/toxics12110831

**Published:** 2024-11-20

**Authors:** Hao Zhan, Cheng-Zhi Li, Yi Kang, Xiao-Zhang Yu

**Affiliations:** College of Environmental Science & Engineering, Guilin University of Technology, Guilin 541004, China; 1020230520@glut.edu.cn (H.Z.); 1020190262@glut.edu.cn (C.-Z.L.); 1020210405@glut.edu.cn (Y.K.)

**Keywords:** metal tolerance proteins, mineral compounds, Ga accumulation, rice seedlings

## Abstract

Mineral elements typically act as transported substrates for metal tolerance proteins (MTPs). The chelation of MTPs with heavy metal ions is a suggestive detoxification pathway in plants; therefore, the trade-off between transporting mineral elements and chelating excess toxic metal ions is inevitable. Gallium (Ga) is an emerging pollutant associated with high-tech industries. This study investigated the impact of Ga stress on MTPs, subsequently altering the transport and distribution of mineral elements. Gallium exposure reduced rice seedling biomass, with roots accumulating more Ga than shoots. Ga stress also changed the rice plants’ subcellular mineral element distribution. PCR assays showed that Ga stress negatively affected all genes belonging to the Mn group, except OsMTP9. While Mn accumulation in the rice cellular compartments did not respond positively to Ga stress, OsMTP8, OsMTP8.1, OsMTP11, and OsMTP11.1 were found to be intimately connected to Mn transport and repressed by increased Ga accumulation in roots. Mg and Cu accumulated in the cytosol and organelles of Ga-treated rice plants, while OsMTP9 expression increased, demonstrating its importance in transporting Mg and Cu. A positive link between Ga stress and Zn accumulation in the cytosol and organelles was found, and OsMTP7 and OsMTP12 expression was positive, suggesting that Ga stress did not impair their Zn transport. Notably, Ga exposure down-regulated Fe-transporting OsMTP1 and OsMTP6, wherein the subcellular concentrations of Fe showed negative responses to Ga accumulation. These findings provide valuable insights into elucidating the roles of OsMTPs in Ga tolerance and the transport of these mineral elements.

## 1. Introduction

Divalent mineral compounds, such as Mg, Cu, Fe, Mn, and Zn are essential nutritional elements that support plant growth and development throughout their entire life cycle [1,2]. These compounds often serve as catalytic or structural cofactors in various enzymes and regulatory proteins that are crucial for performing biological and physiological reactions [3]. Although important for many biochemical activities, an imbalance of these elements in plant tissues can disturb and harm plant functions, resulting in decreases in agricultural productivity and the degradation of quality [4]. Plants have evolved homeostasis mechanisms to regulate the uptake, distribution, and storage of these compounds [5,6]. Maintaining appropriate levels of these elements in plant cells and distributing them correctly within intracellular compartments is essential for healthy plants [7,8]. Diverse transporters are responsible for moving various mineral substrates in plants, ensuring the initiation of many reactions and meeting specific requirements [9]. Among these transporting proteins, cation diffusion facilitators (CDFs) play a crucial role in metal tolerance and homeostasis [10,11]. In plants, CDF proteins are known as metal tolerance proteins (MTPs). Phylogenetic analysis groups these into three categories, namely Zn-MTP, Zn/Fe-MTP, and Mn-MTP, based on their substrate specificity [12,13]. Besides Zn, Mn, and Fe, other divalent cations such as Cu, Mg, Co, Ni, and Cd also serve as transported substrates for MTP proteins in plants [1,6,12,14,15]. In fact, members of the MTP family are located in different membranes responsible for transporting metal ions from the cytoplasm to extracellular compartments or other organelles [7,16].

Heavy metal pollutants, by-products of the industrial revolution, have caused unavoidable damage to the biosphere, severely impacting the natural environment and living organisms [6]. Plants, due to their sessile nature, are particularly susceptible to heavy metal bioaccumulation [17]. Mostly, the existence of heavy metals above tolerance levels has a detrimental impact on plant growth, development, nutritional equilibrium, and enzymatic functions, as well as physiological, biochemical, and molecular processes [17,18,19,20,21]. Most plants have developed natural mechanisms to cope with heavy metal stress. For instance, compartmentalizing heavy metals into vacuoles or the cell wall isolates these toxic ions from sensitive cellular processes, reducing their phytotoxicity [20,22]. Additionally, endogenous enzymatic and non-enzymatic systems are the commonly reported detoxification mechanisms that are highly activated to mitigate oxidative damage caused by the unchecked production of reactive oxygen species (ROS) in plants [17,20,23]. Furthermore, the presence of metal-binding ligands or proteins has been observed as a tolerance mechanism for heavy metal stress [2,24,25]. Gallium (Ga), an element that occurs naturally in low quantities in ecosystems, has experienced an increase in its concentrations as a result of human activity, including the production of semiconductor wafers, solar photovoltaic cells, and light-emitting diodes, during the last two decades [26,27]. China has been reported to provide approximately 84% of the total Ga production worldwide [28]. Consequently, industrial activities and large volumes of e-waste have significantly increased Ga presence in agricultural systems, eventually entering the food chain [29]. Several analyses have already documented the adverse impacts of Ga exposure on the growth and development of plants [29,30,31,32]. For instance, several traits associated with root system architecture demonstrated that rice root system architecture altered in response to Ga stress [32]. Also, the Ga contamination altered the uptake and translocation of nutrients in plants [28,29,30]. Additionally, the accumulation of Ga in rice grains revealed its penetration into the food chain, and this might cause several adverse effects on humans [28,29].

A complicated and multifaceted interaction exists between heavy metal stress and mineral compounds in plants. The synergistic effects of heavy metal ions and mineral elements not only intensify stress but also have adverse effects on the absorption, transport, and distribution of vital minerals in plant tissues [6,17,33]. For instance, high concentrations of divalent heavy metal ions such as Cd and Ni can compete with Zn and Ca for uptake, leading to deficiencies in these essential minerals [34,35]. Additionally, heavy metals can interfere with the movement and dispersion of mineral nutrients in plants, therefore changing the balance of nutrients and consequently impeding plant growth and development [36,37]. Previous studies have shown that Cr(III) exposure not only altered the distribution of Fe, Zn, and Mn in rice plants but also caused changes in the gene expression of OsMTP, suggesting binding interactions between Cr(III) ions and MTP proteins in rice [38]. Due to the similar chemical properties of Ga(III) and Cr(III), we hypothesized that the binding interactions between Ga(III) ions and OsMTP might affect and/or disturb the expression of OsMTP, consequently influencing the transport and distribution of mineral elements in rice plants. While Ga is not extensively found in the natural environment, the exponential expansion of high-tech sectors is generating apprehensions regarding its impact on crops [29]. Therefore, the following studies were conducted: (1) structural characterization of the rice MTP gene family; (2) investigation of the interaction between Ga(III) ions and individual OsMTP proteins using molecular docking tools; (3) expression analysis of the rice MTP genes in different rice tissues under Ga stress; (4) examination of the subcellular distribution of Ga and mineral elements in rice plants under Ga stress; and (5) selection of the key OsMTP genes activated in interactions between Ga stress and mineral elements. These investigations are essential for elucidating the function of OsMTP proteins in controlling and maintaining the movement, equilibrium, and dispersion of mineral nutrients in rice plants after exposure to Ga stress.

## 2. Materials and Methods

### 2.1. Plant Materials and Treatments

The experimental rice seeds (*Oryza sativa* L. cv. XZX45) utilized in this work were supplied by the Hunan Academy of Agricultural Sciences in China. Following a 24-h immersion in distilled water, the rice seeds were subsequently sown in pots containing river sand. The containers were subsequently positioned inside a climate-controlled chamber (LRH-550GSI, Shaoguan Taihong Medical Appliance Co., Ltd., Shaoguan, China), wherein the temperature was regulated at 25 ± 0.5 °C, the relative humidity was maintained at 65 ± 2%, and the plants were exposed to continuous illumination (20,000 lux). Over the time of growth, the plants were irrigated with a modified 8692 nutrition solution to guarantee their access to all essential nutrients [21]. After 16 days, rice seedlings were collected and washed with water and an ionic removal buffer. Finally, ten seedlings with a comparable size were exposed to a 50 mL solution containing 0, 3.0, 50.0, and 180.0 mg Ga/L for a two-day period. These concentrations were selected to reflect three distinct effective concentrations of Ga, namely EC_20_, EC_50_, and EC_75_, showing the minimum, medium, and maximum effective concentrations to suppress the relative growth rate of rice seedlings [32]. Gallium nitrate (Ga(NO_3_)_3_.9H_2_O) of analytical grade with ≥99.5% purity was obtained from Shanghai Macklin Biochemical Co., Ltd. (Shanghai, China) and other chemicals of analytical grade purity were purchased from Sinopharm Chemical Reagent Co., Ltd. (Shanghai, China). To prevent excessive nitrate supply, the Ga-contained solution was not enriched with nitrate from the modified 8692 nutritional solution. Each treatment concentration was conducted in 4 individual replicates.

### 2.2. Subcellular Distribution of Ga and Divalent Cations

The concentrations of Ga and divalent cations (Fe, Cu, Mg, Mn, and Zn) in subcellular compartments, i.e., cell wall, cytosol and organelle, was determined using the gradient centrifugation method [20]. After Ga exposure, roots and shoots were homogenized with 10 mL grinding medium [MES-Tris buffer (50 mM, pH 7.8) + sucrose (0.25 mM) + MgCl_2_ (1 mM) + cysteine (10 mM) at 4 °C] using a freezing mortar and pestle. The differential centrifugation procedure was described according to the protocol described in the Supplementary Materials M1. Finally, three subcellular fractions were all digested using the digestion solution of 4:1 HNO_3_-HClO_4_ and measured by ICP-AES (PerkinElmer Optima 7000 DV, Shelton, CT, USA). The instrument was set according to the following subsequent conditions: a radio frequency power of 1.3 kW, a plasma gas flow rate of 13 L/min, and an auxiliary gas flow rate of 0.2 L/min. For the element measurement, Mg, Zn, Fe, Mn, Cu, and Ga at 285.213, 206.200, 231.617, 257.625, 327.409, and 417.206 nm were used, respectively. The detection limits for these elements were 0.23, 0.64, 0.49, 0.17, 0.06, and 0.06 μg/L, respectively.

### 2.3. Phylogenetic Analysis, Gene Structure, Transmembrane Structure, and Cis-Elements of MTPs

Based on our previous work, 10 isoforms, i.e., *OsMTP1*, *OsMTP5*, *OsMTP6*, *OsMTP7*, *OsMTP8*, *OsMTP8.1*, *OsMTP9*, *OsMTP11*, *OsMTP11.1*, and *OsMTP12* from the rice MTP family are identified [38]. The phylogenetic relationships of the MTP proteins from rice were inferred using the MEGA7.0.18 software with the neighbor-joining method and validated with 1000 bootstrap replicates.

The protein sequences of rice MTPs were downloaded from the database RAP-DB (http://rapdb.dna.affrc.go.jp/, accessed on 10 August 2024). Then, the domains and functional sites in each rice MTP gene were examined with Pfam tool (http://pfam.xfam.org/). Finally, intron–exon structures of all *OsMTP* genes were drawn and visualized using the Exon-Intron Graphic Maker 4 (http://www.wormweb.org/exonintron accessed on 10 August 2024).

The Locus ID of 10 rice MTPs were obtained from the RAP-DB to search the Accession of their respective MTPs from the database Uniport (https://www.uniprot.org/, accessed on 12 August 2024). Then, the online program Protter 1.0 (http://wlab.ethz.ch/protter/start/ accessed on 12 August 2024) was used to analyze and visualize the cation efflux transmembrane domain.

Promoter sequences (~2000 bp) of *OsMTP* genes were obtained from the rice database RAP-DB, and *cis*-elements were identified by the online program Plant CARE (http://bioinformatics.psb.ugent.be/webtools/plantcare/html/, accessed on 16 August 2024).

The MEME 5.5.5 program (https://meme-suite.org/meme/tools/meme, accessed on 12 August 2024) was used to analyze motifs in all rice MTP gene sequences. Then, motif diagrams were drawn using the TBtools 2.097 software [39].

Subcellular localization of all *OsMTP* genes was analyzed from the online database of Uniport and Protter 1.0.

The co-expression network analysis was conducted by the STRING program (https://version-10-5.string-db.org/, accessed on 16 August 2024), and the protein–protein interaction (PPI) networks (combined score > 0.4) were constructed. Then, the figure (resolution = 0.8) with the higher visualization was performed by the program Gephi 0.9.2.

### 2.4. Molecular Docking of Metal Tolerance Proteins

All isoforms of MTPs were used to analyze the binding potential with Ga^3+^ ions. The Hex software (http://hex.loria.fr/, version 8.0.0, accessed on 16 August 2024) was used for protein docking to estimate the possible interaction of Ga^3+^ with different isoforms to form rice MTP proteins. Retrieval of Ga^3+^ (CID: 105,145) for docking was conducted by the chemical database PubChem (https://pubchem.ncbi.nlm.nih.gov/compound/105145, accessed on 16 August 2024). 

The BIOVIA Discovery Studio program (https://www.3ds.com/products/biovia/discovery-studio, accessed on 18 August 2024) was used to analyze the binding bonds formed by Ga^3+^ ions and specific atoms located at the AA residues. The complexes of Ga^3+^ ions with AA residues from different rice MTP isoforms were visualized by the online program PyMOL 3.0 (https://pymol.org/, accessed on 18 August 2024). The docked complexes with the binding energy were judged by the absolute values of binding energy (kcal/mol), wherein the higher absolute values of binding energy indicate the more effective docking process [40]. 

### 2.5. Analysis of Gene Expression

RNA isolation and purification were described previously [21]. The detailed procedure is provided in the Supplementary Materials M2. The transcriptional abundance of 10 genes from the rice MTPs family in rice tissues at various concentrations of Ga was quantified using the RT-qPCR test. The sequence of primers and the cycling conditions of the PCR test is given in Supporting Information Table S1. The RT-qPCR was performed by the 7500 Fast RT-qPCR system (Applied Biosystems, Life Technologies, Foster City, CA, USA) and SYBR green chemistry. *OsGAPDH* (LOC_Os08g03290.1) was used as the housekeeping gene. The standard 2^−ΔΔCT^ method was used to calculate the relative expression of the targeted genes [41].

### 2.6. Data Analysis

Each of the studies was conducted with four biological replicates, and the results are shown as the mean ± standard deviation (SD). The study utilized the Tukey multiple comparison test to establish the substantial difference between the control and treatment groups at a significance level of *p* < 0.05. 

## 3. Results

### 3.1. Gene Structure and Conserved Domains of Rice MTPs

**Figure 1:**
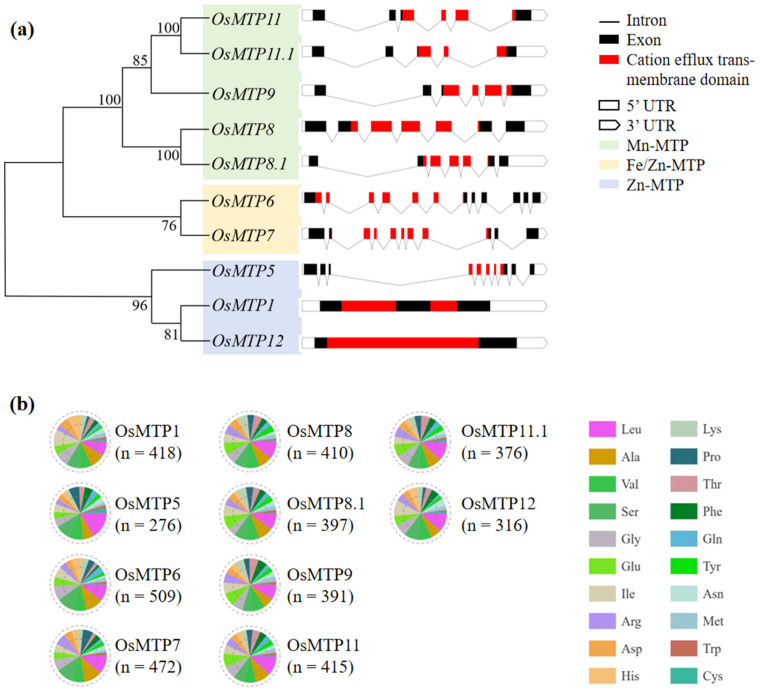
Phylogenetic relationship; the exon–intron structure (**a**) and amino acid composition (**b**) of rice MTP proteins.

Based on the phylogenetic analysis of rice MTPs (Figure 1a), 10 isogenes were grouped in 3 clusters, i.e., Zn-MTP (*OsMTP1*, *OsMTP5*, and *OsMTP12*), Fe/Zn-MTP (*OsMTP6* and *OsMTP7*), and Mn-MTP (*OsMTP8*, *OsMTP8.1*, *OsMTP9*, *OsMTP11*, and *OsMTP11.1*). As shown in Figure 1a, all rice MTP genes had different number and length of exon or intron. For instance, in the cluster of Zn-MTP, *OsMTP5* had 6 exons and 9 introns, wherein *OsMTP1* and *OsMTP12* only had 3 and 2 exons, respectively. In the cluster Fe-Zn MTP, *OsMTP6* had 7 exons and 11 introns, while *OsMTP7* had 5 exons and 10 introns. In the cluster Mn MTP, all isogenes of OsMTPs had 4 exons, but the lengths were quite different, wherein they contained 4–6 introns.

### 3.2. Amino Acid Composition of Rice MTPs

Amino acid composition analysis revealed that the species and numbers of AAs were different among 10 *OsMTP* isogenes (Figure 1b). For instance, the total AA residues in 10 MTP isoforms were 418, 276, 509, 472, 410, 397, 391, 415, 376, and 316, respectively, wherein the most abundant AA residues in 10 MTP isoforms were Ile (42, 10.0%), Leu (46, 16.7%), Ala (56, 11.0%), Leu (53, 11.2%), Leu (44, 10.7%), Leu (40, 10.1%), Leu (36, 9.2%), Leu (50, 12.0%), Leu (43, 11.4%), and Leu (36, 11.4%), respectively. 

### 3.3. Motif Analysis and Transmembrane Structure of Rice MTPs

**Figure 2:**
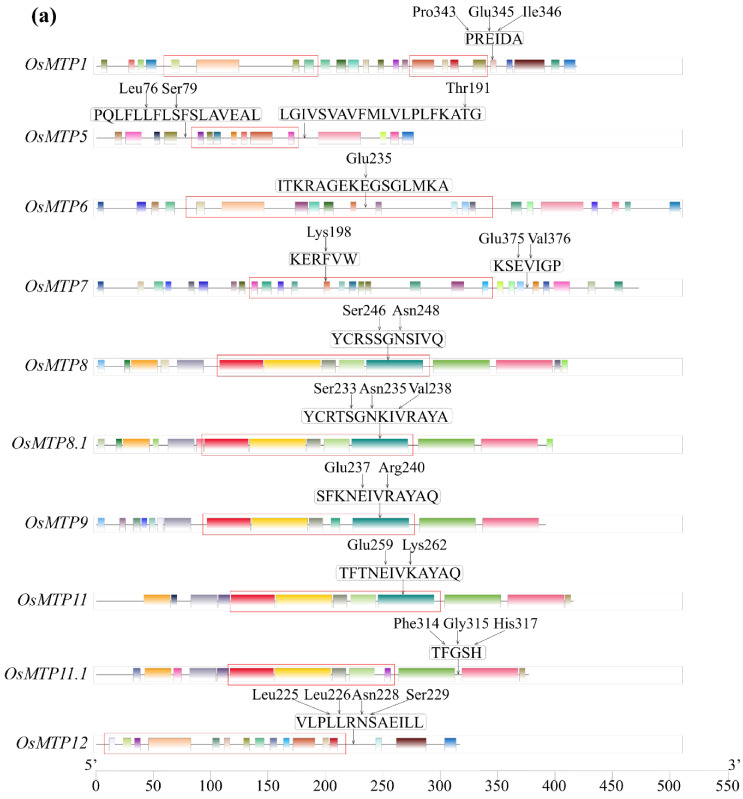

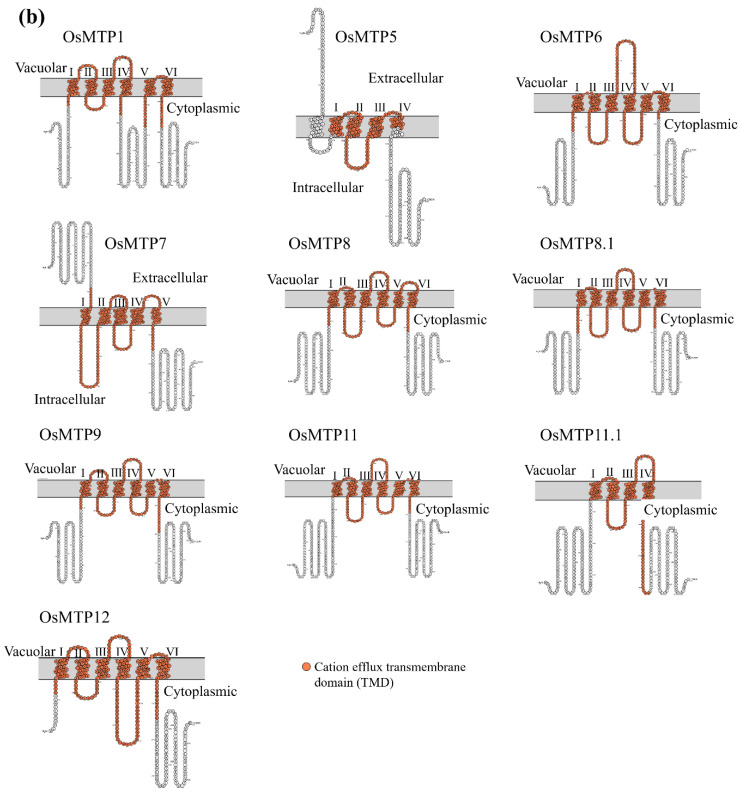
The motif composition (**a**) and the cation efflux transmembrane domains (TMDs) (**b**) of rice MTP proteins. Arrows in Figure 2a refer to binding sites, while red square frames in Figure 2b represent TMDs.

The conserved motifs of all rice MTP proteins were predicted using the MEME program (Figure 2a). Obviously, the number, type, and order of motifs were quite different between individual rice MTP proteins. Even with the isoprotein belonging to the same cluster, significant differences in number, types and order of motifs were observed. For instance, in the cluster of Zn-MTP, 24, 15, and 15 motifs were identified in OsMTP1, OsMTP5, and OsMTP 12, respectively. It is noticed that a lesser number of motifs was observed in the cluster of Mn-MTP, wherein 14, 14, 14, 12, and 13 motifs were identified in OsMTP8, OsMTP8.1, OsMTP9, OsMTP11, and OsMTP11.1, respectively. Additionally, more motifs were observed in OsMTP6 (21 motifs) and OsMTP7 (28 motifs).

The cation efflux transmembrane domains (TMDs) in all rice MTP isoproteins were verified by the program Protter 1.0. As shown in Figure 2b, there were 4–6 TMDs in rice plants located at different sites of the protein sequences of rice MTPs, wherein four TMDs were located at the MTP1 protein sequence of 54–187, and four TMDs were distributed at the MTP1 protein sequence of 273–338. MTP5 and MTP11 had four TMDs located at the different sites of the protein sequences of 73–197 and 125–319, respectively. MTP7 had five TMDs located at the sequence of 129–345. The other MTPs had six TMDs located at the different sites of the protein sequences of OsMTP6 (76–298), OsMTP8 (109–311), OsMTP8.1 (96–298), OsMTP9 (100–299), OsMTP11.1 (124–283), and OsMTP12 (12–234), accordingly. We also noticed that eight rice MTP genes including *OsMTP1*, *OsMTP6*, *OsMTP8*, *OsMTP8.1*, *OsMTP9*, *OsMTP11*, *OsMTP11.1*, and *OsMTP12* were located at the vacuole membrane, while the other two MTP genes (*OsMTP5* and *OsMTP7*) were distributed at the membrane.

### 3.4. Promoter Analysis of MTP Genes in Rice

The 2 kb length promoter regions were selected for analyzing *Cis*-regulatory elements (CREs) in OsMTP genes. Thirty-three common CREs were identified in the promoter of *OsMTP* genes, and these can be categorized into six groups, i.e., light responsive, phytohormone responsive, environmental stress responsive, general regulatory elements, regulation of plant development, and binding responsive, respectively (Table 1). The overall frequency of these CREs in the upstream region of individual *OsMTP* genes was variable, wherein the descending order of the frequency was *OsMTP9* (21 CREs), *OsMTP11.1* (20 CREs), *OsMTP1* (20 CREs), *OsMTP11* (19 CREs), *OsMTP8.1* (18 CREs), *OsMTP7* (17 CREs), *OsMTP5* (15 CREs), *OsMTP6* (14 CREs), *OsMTP8* (13 CREs), and *OsMTP12* (12 CREs), and the detailed information is provided in the Supplementary Materials Table S3. Conversely, six CREs belonging to the groups of light responsive and phytohormone responsive were unique to 5 *OsMTP* genes (*OsMTP1*, *OsMTP7*, *OsMTP8*, *OsMTP9*, and *OsMTP11*) (Table 2). Interestingly, tw out of six unique CREs were present in *OsMTP7*, whereas the other four genes only contained one unique CRE.

### 3.5. Molecular Interaction Between MTPs and Ga^3+^ Ions

**Figure 3:**
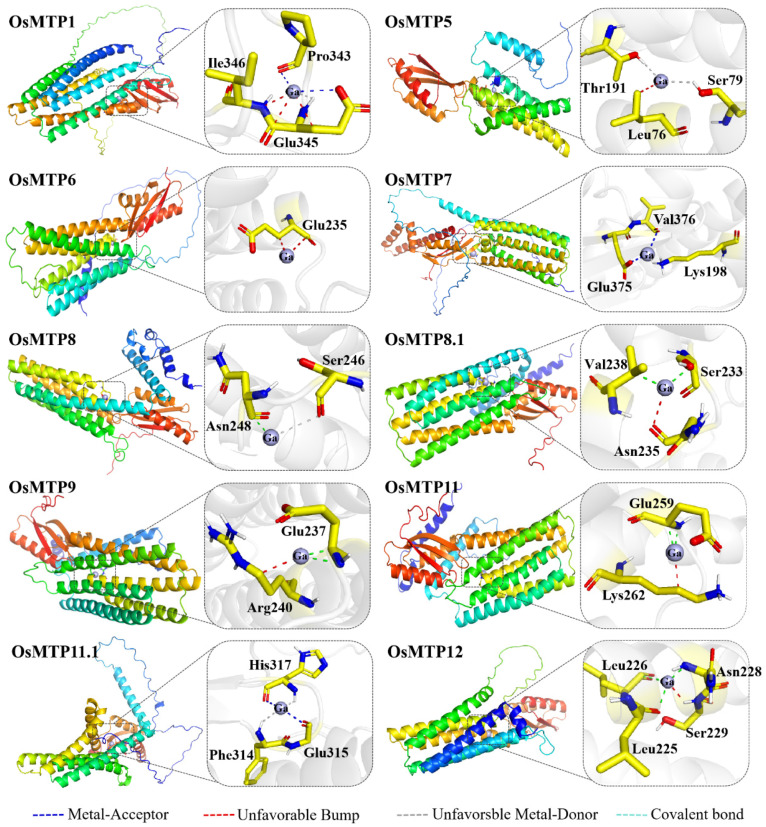
Binding sites of rice MTP proteins with Ga ions.

The Hex program was used to stimulate the interaction between Ga^3+^ ions and the OsMTP protein and identify the possible protein-compatible binding sites. Figure 3 shows the interaction sites of 10 individual OsMTP proteins with Ga^3+^ ions. One to four binding sites located at different amino acid residues were identified in individual OsMTP proteins. For instance, Ga^3+^ ions bind to Pro (343), Glu (345), and Ile (346) amino acid residues of OsMTP1, and three different types of bonds, including metal acceptor, unfavorable metal-donor, and unfavorable bump are formed between the Ga^3+^ ions and the amino acid residue at OsMTP1, wherein these types of bonds have weaker affinity potential compared with the covalent bond. Specially, the carbonyl (–C=O) of Pro (343) located at C-CA-CB-CG-CD of OsMTP1 provides a site for binding Ga^3+^ ions through metal-acceptor interaction, while the carboxyl (–COO-) of Glu (345) located at the C-CA-CB-CG-CD of OsMTP1 is the binding site for Ga^3+^ ions through metal acceptor interaction. The detailed information of binding sites at the specific amino acid residues of individual OsMTP are given in Table 3.

### 3.6. Subcellular Distribution of Ga, Mg, Cu, Fe, Mn, and Zn in Rice Tissues

**Figure 4:**
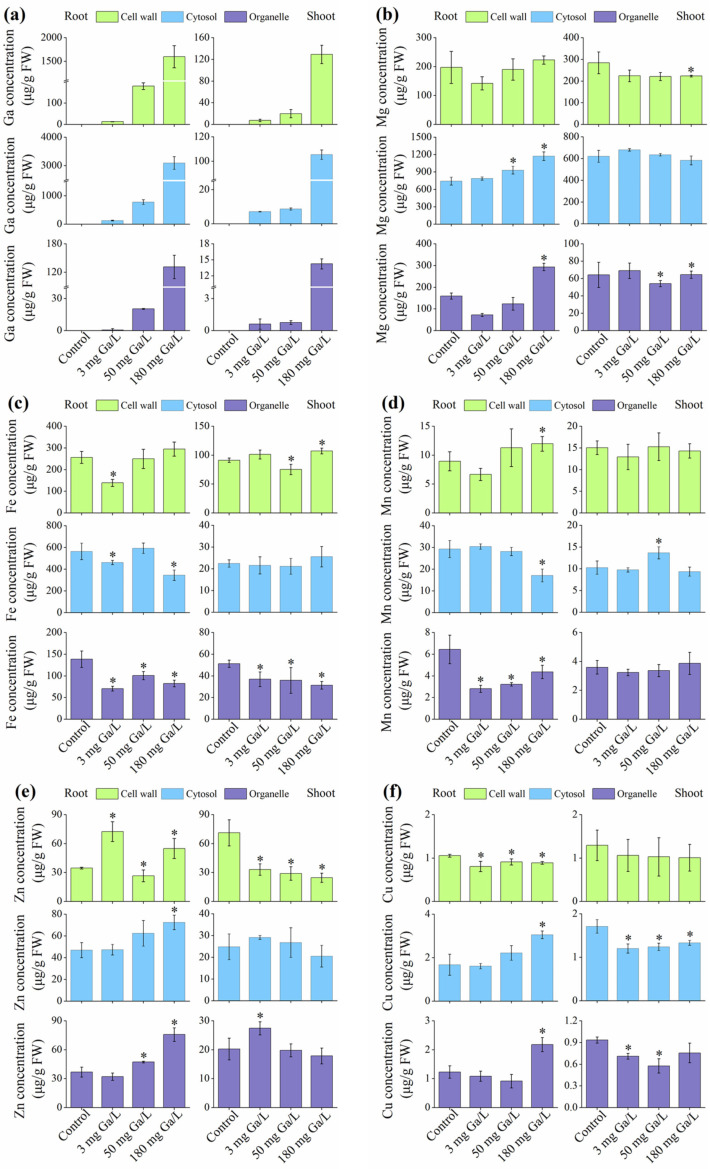
Subcellular concentrations (μg/g FW) of Ga (**a**) and mineral elements, including Mg (**b**), Fe (**c**), Mn (**d**), Zn (**e**), and Cu (**f**) in rice tissues. Values are the mean of four independent biological replicates ± standard deviation. NA denotes concentrations below the limit of Ga detection. The asterisk (*) refers to the significant difference between Ga treatments and control.

With increasing Ga concentrations, the proportions of Ga in subcellular compartments displayed a notable accumulation trend (Figure 4). Significant differences were noted in the distribution of Ga among the various subcellular compartments of shoots and roots. The rice plant roots exhibited the greatest proportion of Ga in the cytosol, which was then observed in the cell wall and organelles (Figure 4a). The cytoplasm of rice plant roots had the largest Ga fraction (Figure 4a). The cell wall of the shoots had the greatest Ga percentage, which was followed by the cytosol and organelles.

However, the distribution of Mg, Fe, Mn, Zn, and Cu in the various subcellular compartments of rice tissues was also markedly changed by Ga exposure. In the roots, the largest fraction of Mg was detected in the cytosol, while the difference in the fractions of Mg in the cell wall and organelles was negligible (Figure 4b). In the shoots, the largest fraction of Mg was also detected in the cytosol, followed by the cell wall and organelles. The largest fraction of Fe in roots was also detected in the cytosol, followed by the cell wall and organelles (Figure 4c), wherein the largest fraction of Fe in shoots was also detected in the cell wall, followed by organelles and the cytosol. The largest fraction of Mn in roots was also detected in the cytosol, followed by the cell wall and organelles (Figure 4d), wherein the largest fraction of Mn in shoots was also detected in the cell wall, followed by the cytosol and organelles. The largest fraction of Zn in roots was also detected in the cell wall, followed by the organelles and cytosol (Figure 4e), wherein the largest fraction of Zn in shoots was also detected in the cell wall, and difference between the cytosol and organelles. The largest fraction of Cu in roots was also detected in the cytosol (Figure 4f), followed by the organelles and cell wall, wherein the largest fraction of Cu in shoots was also detected in the cytosol, followed by the cell wall and organelles. The detailed fractions of Ga and mineral elements in different subcellular compartments of rice tissues is provided in Supporting Information Figure S1.

### 3.7. Gene Expression of MTPs to Ga Exposure in Rice Tissues

**Figure 5:**
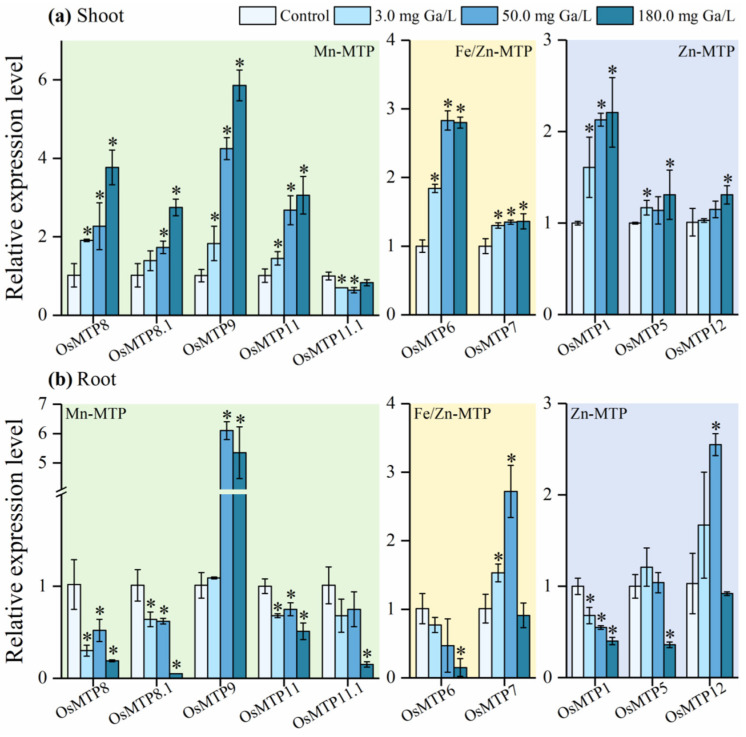
The relative expression of individual MTP genes in rice tissues under Ga stress. Values are the mean of four independent biological replicates ± standard deviation. The asterisk (*) refers to the significant.

The expression abundance of the 10 *OsMTP* genes was evaluated in shoots (Figure 5a) and roots (Figure 5b) of Ga-treated rice seedlings. Only three genes, i.e., *OsMTP7*, *OsMTP9*, and *OsMTP11* were up-regulated in roots at Ga treatment concentrations compared to the control, while the other 7 *OsMTP* genes showed significantly negative responses to Ga exposure. Only *OsMTP11.1* was significantly down-regulated in shoots of Ga-treated rice plants, but the remaining *OsMTP* genes were significantly down-regulated compared to the control. It is noticed that *OsMTP7*, *OsMTP9*, *OsMTP12*, and *OsMTP11.1* showed a consistent expression pattern between roots and shoots, and other *OsMTP* genes demonstrated completely different expression patterns.

### 3.8. Integration of Gene Expression, Mineral Elements, and Ga Accumulation

**Figure 6:**
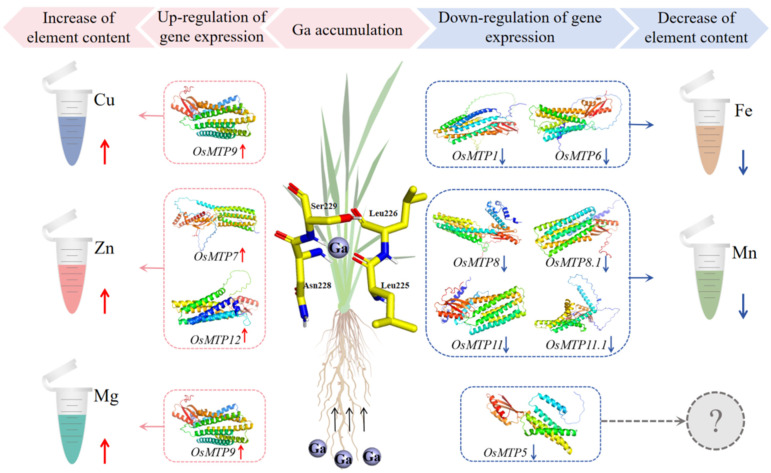
The interconnection between Ga accumulation, concentrations of mineral elements, and expression of individual rice OsMTPs in rice roots.

In this regard, the chelation of OsMTP with Ga^3+^ ions is a detoxification process to reduce the phytotoxicity of Ga stress to rice plants. However, the role of OsMTP in transporting mineral elements was never taken lightly. Therefore, the trade-off between transporting mineral elements and chelating Ga^3+^ ions was unavoidable. We noticed that Ga accumulation in rice plants significantly altered the expression patterns of rice *OsMTP* genes and the distribution of mineral elements in subcellular compartments in rice tissues. The interconnection between Ga accumulation, concentrations of mineral elements, and individual rice OsMTPs is presented in Figure 6.

### 3.9. Rice MTP Social Network

**Figure 7:**
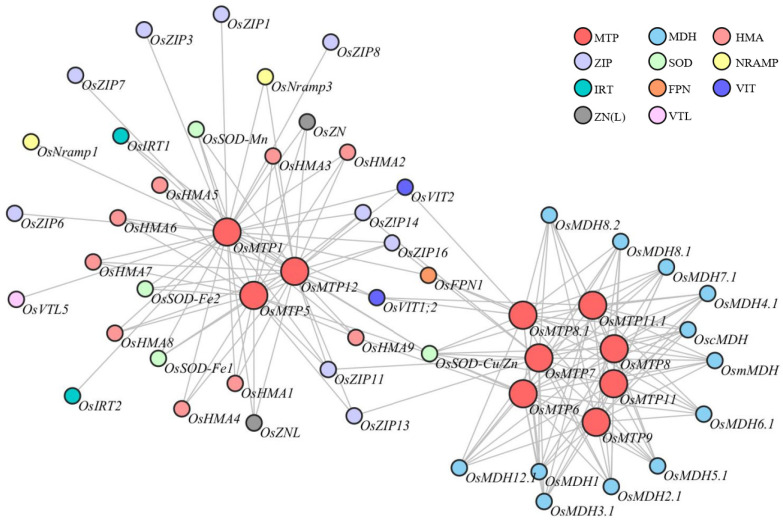
Social networks of rice MTP genes.

Several types of metal transport proteins were clarified in plants, including the heavy metal ATPase family (HMAs), MTPs, natural resistance-associated macrophage proteins (NRAMPs), the iron-regulated transporter (IRT), the ferroportin (FPN), the vacuolar iron transporter (VIT) and Zn-regulated transporter, and the Fe-regulated transporter-like family (ZIP) [42,43,44,45,46]. Mostly, plants’ physiological processes were chiefly regulated by various genes through the coordinated and interacted network. Herein, the co-expression network of individual *OsMTP* genes were conducted by the STRING program (Figure 7). The interactome analysis revealed that *OsMTP1* highly interacts with seven different types of genes; however, major interactive genes are associated with metal transport, such as *OsFPN*, *OsHMAs*, *OsNRAMP*, *OsIRT*, *OsVIT*, and *OsZIP*, wherein *OsMTP1* is highly activated with *OsSOD* genes. It is also noticed that *OsMTP12* highly interacts with *OsHMAs*, *OsIRT*, *OsVIT*, *OsNRAMP*, *OsZIP*, and *OsSOD*. However, somewhat interesting results were obtained for *OsMTP6*, *OsMTP7*, *OsMTP6*, *OsMTP8*, *OsMTP8.1*, *OsMTP9*, *OsMTP11*, and *OsMTP11.1*, wherein the major interactive genes of these genes are highly related to malate dehydrogenase rather than metal transport. The detailed information of the interactive genes of individual OsMTP is provided in Supporting Information Table S4.

## 4. Discussion

A multitude of investigations have been undertaken to elucidate the possible processes linked to the uptake, retention, movement, phytotoxicity, and elimination of heavy metals in various plant species [45]. In this study, the accumulation of Ga in rice plants was evident, showing a dose-dependent manner, which is consistent with the previous works [32]. Additionally, approximately 88.5% (SD: 5.09, No: 3) were detected in the roots of rice seedlings after two days of exposure. A similar finding was also made by Syn et al. [47], whereby notably greater levels of Ga were observed in the roots than in the shoots of rice seedlings subjected to Ga treatments with different dosages. Moreover, subcellular distribution analysis showed that the distribution of Ga in different compartments of roots and shoots was different. In roots, the fraction of Ga in the cytosol showed a decreasing pattern with an increase in Ga concentration, while increasing concentrations of Ga increased the fraction of Ga in the cell wall. In shoots, the highest fraction of Ga was detected in the cell wall. These findings indicated that the segregation of Ga into the cell wall was a proactive survival mechanism to deal with Ga-induced stress in rice plants [20,22]. Moreover, Ga exposure significantly altered the subcellular distribution of measured nutrient elements in rice tissues. In this regard, a correlation analysis between all measured mineral elements and Ga concentrations in different subcellular compartments was conducted, and the correlation coefficient (*R* ≥ 0.90 or *R* ≤ -0.90) judged the significant correlation (results are given in the Supplementary Materials Figure S2). We noticed that the concentrations of all measured mineral elements in the cell wall in both roots and shoots did not show significant correlation (*p* > 0.05) to Ga concentration. Only the concentrations of Mg, Zn and Cu in the organelle compartment showed positive correlation (*p* < 0.05) to Ga concentration, while the correlation between these elements in shoots and Ga was not significant (*p* > 0.05). It is noteworthy that the concentrations of all measured mineral elements in cytosol in rice roots showed significant correlations to the concentrations of Ga in cytosol, wherein Ga concentrations in the cytosol compartment had a positive relationship (*p* < 0.05) with Mg, Zn, and Cu, and a negative correlation (*p* < 0.05) with Mn and Fe. The studies revealed that exposure to Ga had a substantial impact on the distribution of mineral elements in various subcellular compartments by employing distinct regulatory mechanisms.

In plant cells, metal-associated transporters are integral membrane proteins that regulate metal homeostasis, control the acquisition of metal ions into plants’ cells, coordinate the distribution of these compounds to appropriate organelles, and prevent and/or mediate negative consequence due to excess or deficiency of these elements [45,47]. It is known that divalent ions have higher redox potential and can bind amino acids [48]. Moreover, the metal-binding domains located at the metal-associated proteins become a gathering site for metal ions; consequently, the metal-amino acid complex is formed and transports the membrane of cells into extracellular space or into intracellular organelles [42,47,48]. During the complex process between metal ions and amino acids located at the metal-binding domains of metal-associated proteins, amino acid residues mainly serve as electron donors [48]. Additionally, the metal-binding domains with a highly conserved sequence should be rich in Cys, Asp, Glu, His, and Met amino acid residues [47]. This is because Cys and Asp residues have negative charge, while Glu and His residues have hydrophilic and polar properties [49]. In fact, these metal-associated transporters have been categorized into channels, carriers, and pumps [50,51]. Among these transporters, MTPs are identified as important metal transporting proteins. Assuredly, most plants’ MTPs have 4–6 TMDs and a signature N-terminal amino acid sequence [42], which are the translocation sites for metal ions, mostly likely mediated by a complex of specific amino acid residues located at the TMDs [52]. Rice MTP proteins, except for *OsMTP5* and *OsMTP7* distributed at the membrane, are vacuolar transporters and showed metal transporting potential [53]. In fact, transporting divalent metal ions, i.e., Zn, Fe, Mg, Mn, Cu, Co, and Cd from the cytoplasm to the extracellular or subcellular fractions by MTPs have been observed in various plants [1,6,12,14,15,45]. For instance, *AtMTP1* and *AtMTP3* are responsible for deposition of Zn and Co into vacuole [54,55], and accumulation of Zn into Golgi organelle was initiated by *AtMTP5* [7]. In rice plants, involvement in the transport of Zn, Cd, and Ni is suggestive by overexpression and silencing of *OsMTP1* [10,42]. Also, *OsMTP8*, *OsMTP8.1*, and *OsMTP11* of the tonoplast are activated in Mn deposition into Golgi apparatus in rice [8,13,56,57].

It is known that amino acid residues contain different functional groups, i.e., C-H, -NH_2_, -OH, –C=O, and –COOH, which provide possible binding sites for metal ions [58]. In this study, the binding potential between Ga^3+^ ions and OsMTP proteins was simulated by molecular docking analysis, wherein each OsMTP had its specific binding sites (the detailed information of binding sites with specific functional groups is given in the Supplementary Materials Figure S3). Theoretically, the binding energy serves as an indicator for binding potential. As shown in Table 3, the binding energy of individual OsMTPs with Ga^3+^ ions in descending order is OsMTP11 > OsMTP8 > OsMTP11.1 > OsMTP12 > OsMTP7 > OsMTP1 > OsMTP9 > OsMTP6 > OsMTP8.1 > OsMTP5. Additionally, the binding potential of metal ions with proteins is a key factor for determining the activity and function of proteins or enzymes [59]. For instance, metal ions, such as Hg, Pb, Fe, Cu and Zn, have resulted in a negative impact on the activity of α-amylases through binding with –C=O groups or indole rings of His and Cys residues [60,61]. In fact, the most convincing evidence to describe the strength of affinity potential between metal ions and proteins is the formation of chemical bonds, in which the covalent bonds are generally sturdier than non-covalent bonds [62]. In this study, we noticed that besides covalent bonds, three types of bonds, such as metal acceptors, unfavorable metal-donor, and unfavorable bump were also stimulated during the complex process between the Ga^3+^ ions and amino acid residues of OsMTPs. Moreover, only OsMTP8, OsMTP8.1, OsMTP9, OsMTP11, and OsMTP12 had the covalent bonds formed with Ga^3+^ ions. However, results from PCR tests revealed that the expression abundance of *OsMTP9* and *OsMTP12* in roots showed positive responses to Ga exposure. The motif analysis of amino acid sequences showed that the binding sites of OsMTP12, including Leu (225), Leu (226), Ser (229), and Asn (228), were all distributed at the non-motif zones, which might have other non-defined functions. Therefore, it is not surprising that up-regulation of *OsMTP12* in roots might be an activated survival strategy in rice plants to cope with Ga exposures.

In this study, the responses of *OsMTP11.1* to Ga exposure showed a down-regulation pattern in both roots and shoots, while the other three genes, i.e., *OsMTP7*, *OsMTP9*, and *OsMTP12* were up-regulated in both rice tissues. Importantly, other *OsMTP* genes showed completely different expression responses between roots and shoots, wherein down-regulation in roots and up-regulation in shoots was observed, suggesting different regulating mechanisms imposed by Ga exposure. In fact, a similar conclusion was also reached by Ram et al. [63], by which the expression of most *MTP* genes in rice plants down-regulated in root and up-regulated in shoots. Herein, interests have been generated as to why individual *OsMTP* genes show different responses to Ga stress despite them belonging to the same categories which share a close phylogenetic relationship. It is known that motifs typically exhibit conserved patterns with specific functions, in which they may play a crucial role in gene regulation, protein binding, or other molecular interactions [11,64]. In this study, diverse amino acid residues from OsMTP1 (motif 1), OsMTP7 (motif 35), OsMTP8 (motif 4), OsMTP8.1 (motif 4), OsMTP9 (motif 4), and OsMTP11 (motif 4) were predicated to bind with Ga^3+^ ions at specific motifs, while the binding sites for other OsMTP residues were distributed at non-motif zones. The difference in binding sites at either specific motifs or non-motif zones might cause different genetic responses [6]. Additionally, it was reported that the metal binding site was mainly associated with the zone between TMD 4 and TMD 5 [42]. Combining these results, we noticed that only the binding sites of OsMTP8, OsMTP8.1, OsMTP9, and OsMTP11 with Ga^3+^ ions were distributed at the TMD regions. Detailed information regarding the composition of motifs distributed at the TMD regions and the binding sites of individual rice MTP genes located at specific motifs is provided in Supporting Information Table S2. This was consistent with the analysis of molecular docking. These results suggest that motif 4 of OsMTP8, OsMTP8.1, OsMTP9, and OsMTP11 located at the TMD regions may be targeted for sequence-specific binding sites for specific rice MTP proteins attacked by Ga^3+^ ions.

The analysis of protein structures, specifically gene intron–exon characterizations, is a valuable method for comparing gene functions and determining their phylogenetic relationships. Differences in these protein structures may indicate their distinct roles in the transmembrane transport process in response to various environmental stimuli [65]. In this study, intron–exon characterizations of rice MTP members were conducted. Comparisons of intron–exon structures with respective phylogenetic distribution showed that a significant difference was observed in the group of Zn-MTP, while the other two groups, i.e., Mn-MTP and Fe/Zn-MTP showed similar organization for the phylogenetically closer MTP genes. Evidently, the number of introns in the gene were correlated with gene expression and a loss or gain of homologues [63]. We noticed that *OsMTP1*, *OsMTP5*, and *OsMTP12* categorized in the group of Zn-MTP shared the closest phylogenetic relationship. However, *OsMTP1* and *OsMTP12* did not contain the intron, suggesting their differences in the evolutionary relations with other rice MTP isogenes [66], which suggest their different genetic roles, particularly under environmental stresses. The functional redundancy of three Zn-MTP genes in rice has been proposed [10], which is most likely due to the different in the transmembrane domains. It is known that the vital role of transmembrane domains is to determine the specificity of the MTP proteins for different metal ions, and to avoid excess and deficiency of mineral elements [10,12]. In our PCR test, the expression abundance of *OsMTP1* and *OsMTP5* was down-regulated for Ga exposure, and *OsMTP12* was up-regulated in roots, suggesting these different responsive mechanisms to Ga stress, although they were phylogenetically with the Zn group. Additionally, molecular docking analysis revealed that three members of Zn-MTP had a possible binding site with Ga^3+^ ions; however, the covalent bond was only formed with OsMTP12, while weaker bonds were predicated with OsMTP1 and OsMTP5. Moreover, the only binding site was located at motif 1 of Pro (343), Glu (345), and Ile (346) residues of OsMTP1, while the binding site for *OsMTP12* with Ga^3+^ ions was predicated at the non-motif regions, which might not damage its instinct role for transporting Zn. Such a scenario was also observed for *OsMTP7*, belonging to the Fe/Zn group. Ga exposure increased the transcript abundance of *OsMTP12* and *OsMTP7*, and Zn accumulation in cytosol and organelles in roots, suggesting that Ga stress stimulated their transporting potential for Zn.

It is known that several types of metal-associated transporters have been identified in various plants [45,47]. For instance, Zn homeostasis in Arabidopsis is highly regulated by both *AtHMA2* and *AtHMA4* [67]. In rice, *OsVIT1* and *OsVIT2* modulate Fe and Zn allocation between sources and sink tissues, which may represent a potential strategy for Fe/Zn biofortification in rice grains [68]. Additionally, *OsIRT1*, *OsNRAMP1*, and *OsNRAMP5* show a great contribution to Fe and Mn distribution in rice shoots [69,70,71]. Moreover, the characterization of ZIP protein members in plants implicates Zn absorption and transport [72,73]. Results from the interactome analysis revealed that different groups of rice MTP genes had different interaction partners. For instance, the genes categorized in the Zn-MTP group, including *OsMTP1*, *OsMTP5*, and *OsMTP12* were mainly correlated to *OsHMA*, *OsVIT*, *OsIRT*, *OsNRAMP*, and *OsZIP*. However, the member and number of interaction partners for individual *OsMTP* genes were quite different. It is interesting to notice that the interaction partners for the genes belonging to the Fe/Zn group, i.e., *OsMTP6* and *OsMTP7*, were mainly associated with malate dehydrogenase. A similar result was also predicted for the genes categorized in the Mn-MTP group, which also showed higher interactive relationships with malate dehydrogenase rather than metal transporting proteins. It is known that plants’ physiological activities are mediated and regulated by various genes, wherein the involvement and activation of genes in these processes is never independent, and the coordinated and interactive actions between genes may be a good survival strategy. Evidently, energy demand competition in different physiological processes of plants is a restricting factor to limit plant growth. In this study, the interaction partners of the genes belonging to the Zn-MTP group were metal transporting proteins, in which substrate specificity with less energy demand might become the priority to match metal ions and metal transporting proteins. However, the interaction partners of other rice MTP genes were mainly associated with malate dehydrogenase, which is a key enzyme activated energy metabolism process, suggesting that a trade-off between metal transporting proteins and energy metabolism might be initiated to keep plants’ normal function through balancing mineral element homeostasis in different subcellular compartments.

Integrating TMD analysis, docking stimulation, and motif analysis together, we noticed that the Pro, Glu, Ile, Leu, Ser, Thr, Lys, Val, Asn, Arg, Phe, and His amino acid residues were predicted as the possible binding sites of rice MTP proteins with Ga^3+^ ions. Evidently, the TMD region of metal transporting proteins was the translocation site for metal ions mediated by specific amino acid residues [42,52]. Herein, the Glu, Ser, Lys, Val, Asn, and Arg amino acid residues of rice MTPs were located at the TMD region. Moreover, the Glu, Ser, Lys, Val, and Asn amino acid residues interacted with Ga^3+^ ions through the covalent bond, while the Arg residue with Ga^3+^ ions interacted with the unfavorable bump, which is a weaker bond compared with the covalent bond. Furthermore, the amino acid residues located at specific motifs might be the targeted site for Ga^3+^ ions, wherein motif 4 was identified from *OsMTP8*, *OsMTP8.1*, *OsMTP9*, and *OsMTP11*. Results from PCR tests revealed that the expression abundance of these four genes was significantly down-regulated in roots of Ga-treated rice seedlings, except for *OsMTP9*, which showed significant up-regulation. It is known that the mandatory mission for metal transporting proteins is to transport mineral elements and to balance the homeostasis of these compounds in different organelles and different subcellular compartments as well [42,45,48]. We noticed that the binding energy between Mn^2+^ ions and OsMTP9 is −46.96 kcal/mol, which is much less than the binding energy (−41.94 kcal/mol) between Ga^3+^ ions and OsMTP9 (the detailed information of binding sites is given in Supporting Information Figure S3). Additionally, the same amino acid residues with the same distance of OsMTP9 interacted with Mn^2+^ ions as Ga^3+^ ions. Therefore, it is not surprising that transporting Mn^2+^ ions became the first option for OsMTP9 under Ga stress due to its demand of less energy.

The Ga stress changed the MTP gene expression which subsequently altered the transport and distribution of mineral elements. Among 10 rice MTP genes, four genes, i.e., *OsMTP7*, *OsMTP9*, *OsMTP11.1*, and *OsMTP12* need more attention in future work. In this study, only *OsMTP11.1* negatively responded to Ga exposure in both roots and shoots, suggesting its expression was quite sensitive to the presence of Ga in rice tissues, and higher accumulation of Ga in roots caused much more down-regulation. Additionally, the binding site of OsMTP11.1 with Ga^3+^ ions was located neither at the TMD region nor at the motif region, suggesting that Phe (314), Gly (315), and His (317) residues of OsMTP11.1 were the targeted sites attacked by Ga^3+^ ions. It is known that OsMTP9 belongs to the Mn group, which is capable of transporting Mn. The positive expression of *OsMTP9* to Ga stress and the decrease of Mn in rice suggests its involvement in Mn transport was negligible. It was reported that *AtMTP10* was identified in Mg homeostasis in Arabidopsis [15], which shared the closest phylogenetic relationship with OsMTP9 [38]. In fact, Ga exposure caused Mg accumulation in cytosol and organelles, suggesting that the involvement of *OsMTP9* in transporting Mg was highly activated by Ga exposure. In this study, we also observed a positive relationship between Ga stress and Cu concentrations in cytosol and organelles. It has been reported that the genes, i.e., *AtMTP8* and *AtMTP11* are involved in Cu transport [1,14,16], which are categorized into the Mn-group. Our PCR tests showed that only the *OsMTP9* which is categorized in the Mn-group was significantly up-regulated in Ga-treated rice plants. It was suggested that the involvement of *OsMTP9* in transporting Cu was possible. A mocking analysis revealed that OsMTP12 binded with Ga^3+^ ions to form stable covalent bonds, and the binding region was located neither at the TMD region nor at the motif region. Additionally, the expression abundance of *OsMTP12* in rice tissues after Ga treatments showed significant down-regulation, wherein an accumulation of Zn in cytosol and organelles was evident. This analysis suggested that Leu (225, 226), Asn (228), and Ser (229) residues of OsMTP12 might be the activated sites for Ga^3+^ ions to trigger its capacity for transporting Zn in rice plants. A similar result was also observed in OsMTP7, wherein Lys (198), Glu (375), and Val (376) residues of OsMTP7 were the targeted sites to stimulate transporting Zn by Ga^3+^ ions.

## 5. Conclusions

The presented study indicated that OsMTPs played significant roles in Ga tolerance and mineral element transport in rice. The biomass changes of rice seedlings showed a negative correlation to Ga doses. Ga accumulation was significantly higher in roots than in shoots, leading to a significant alteration in the subcellular distribution of measured mineral elements in rice plants. PCR tests indicated that rice *OsMTP* genes showed different responses to Ga exposure. The negative responses of *OsMTP8*, *OsMTP8.1*, *OsMTP11*, and *OsMTP11.1* due to higher Ga accumulation in rice roots resulted in less accumulation of Mn in rice cellular compartments. Notably, the up-regulation of *OsMTP9* triggered by Ga stress and the accumulation of Mg and Cu in rice tissues suggested its crucial role in transporting Mg and Cu. The accumulation of Zn showed a positive correlation with Ga stress, with the expression of *OsMTP7* and *OsMTP12* showing positive responses, suggesting that Ga stress did not shift their preference for transporting Zn. Additionally, the down-regulation of OsMTP1 and *OsMTP6* due to Ga accumulation decreased Fe transport in rice subcellular compartments. Altogether, Ga stress partly altered OsMTP function through chelation processes, subsequently impacting the transport and distribution of essential mineral elements in rice plants. Furthermore, differentially expressed *OsMTPs* would serve as bioindicators for predicting Ga stress and evaluating the homeostasis of essential mineral elements in rice plants, which significantly influences the tailoring of *OsMTPs* for biofortification in future studies.

## Figures and Tables

**Table 1:** Common putative *cis*-elements identified in the promoter sequences of OsMTP genes.

*Cis*-Element Name	Gene Number	Signal Sequence	Location	Function
Light responsive				
G-box	8	CACGAC	1844	Light responsiveness
Box 4	7	ATTAAT	1131	Light responsiveness
GT1-motif	6	GGTTAA	646	Light-responsive element
Sp1	6	GGGCGG	1882	Light-responsive element
TCT-motif	6	TCTTAC	619	Light-responsive element
TCCC-motif	5	TCTCCCT	245	Part of a light-responsive element
MRE	3	AACCTAA	639	Light responsiveness
GATA-motif	3	AAGGATAAGG	581	Part of a light-responsive element
I-box	3	AAGATAAGGCT	705	Part of a light-responsive element
ATCT-motif	2	AATCTAATCC	773	Light responsiveness
chs-CMA1a	2	TTACTTAA	1139	Light-responsive element
GA-motif	2	ATAGATAA	858	Light-responsive element
AE-box	2	AGAAACAA	189	Part of a module for light response
Phytohormone responsive				
CGTCA-motif	9	CGTCA	262	MeJA responsiveness
TGACG-motif	9	TGACG	406	MeJA responsiveness
ABRE	8	ACGTG	1535	Abscisic acid responsiveness
TCA-element	4	CCATCTTTTT	805	Salicylic acid responsiveness
TGA-element	4	AACGAC	480	Auxin-responsive element
P-box	3	CCTTTTG	863	Gibberellin-responsive element
TATC-box	2	TATCCCA	610	Gibberellin responsiveness
Environmental stress responsive				
ARE	9	AAACCA	1457	Anaerobic induction
GC-motif	7	CCCCCG	1879	Anoxic-specific inducibility
TC-rich repeats	5	GTTTTCTTAC	835	Defense and stress responsiveness
circadian	4	CAAAGATATC	1258	Circadian control
LTR	4	CCGAAA	1954	Low-temperature responsiveness
MBS	4	CAACTG	320	Drought inducibility
General regulatory elements				
CAAT-box	10	CCAAT	957	Core enhancer element
TATA-box	10	TATA	1371	Core promoter element
A-box	8	CCGTCC	751	*Cis*-acting regulatory element
Regulation of plant development				
O_2_-site	5	GATGATGTGG	1634	Zein metabolism regulation
CAT-box	3	GCCACT	1697	Meristem expression
Binding responsive				
AT-rich element	4	ATAGAAATCAA	1815	AT-rich DNA binding protein
CCAAT-box	2	CAACGG	1854	MYBHv1 binding site

**Table 2:** Unique *cis*-elements identified in the promoter sequence of OsMTP gene.

Gene	Cis-Element Name	Signal Sequence	Location	Function
Light responsive				
*OsMTP1*	3-AF1 binding site	TAAGAGAGGAA	384	Light-responsive element
*OsMTP7*	LAMP-element	CTTTATCA	143	Part of a light-responsive element
*OsMTP8*	ATC-motif	AGTAATCT	1046	Light responsiveness
*OsMTP9*	chs-Unit 1 m1	ACCTAACCCGC	1764	Part of a light-responsive element
Phytohormone responsive				
*OsMTP7*	AuxRR-core	GGTCCAT	1965	Auxin responsiveness
*OsMTP11*	GARE-motif	TCTGTTG	681	Gibberellin-responsive element

**Table 3:** The detailed binding information of OsMTP-Ga complex.

MTP Protein	Residues of AAs	Distance (Å)	Binding Functional Group	Possible Bonds	Binding Energy (kcal/mol)
OsMTP1	Pro (343)	3.1	–C=O	Metal acceptor	−42.33
	Glu (345)	2.3	–COO– –C –CB	Metal acceptor Unfavorable bump Unfavorable bump	
	Ile (346)	2.3	–CO–NH	Unfavorable metal donor	
OsMTP5	Leu (76)	3.4	–CA	Unfavorable bump	−31.17
	Ser (79)	2.6	–OH	Unfavorable metal donor	
	Thr (191)	2.6	–OH	Unfavorable metal donor	
OsMTP6	Glu (235)	2.4	–C –CB	Unfavorable bump Unfavorable bump	−41.03
OsMTP7	Lys (198)	2.0	–CO–NH	Unfavorable metal donor	−43.33
	Glu (375)	2.3	–COO-	Metal acceptor	
	Val (376)	2.8	–C=O	Metal acceptor	
**OsMTP8**	Ser (246)	3.4	–C=O	Unfavorable metal donor	−46.09
	Asn (248) *	2.2	–C	**Covalent bond**	
**OsMTP8.1**	Ser (233) *	2.1	–OH	**Covalent bond**	−37.54
	Asn (235)	2.6	–C=O	Unfavorable bump	
	Val (238) *	2.1	–C=O	**Covalent bond**	
**OsMTP9**	Glu (237) *	1.8	–CA –CO–NH	**Covalent bond** **Covalent bond**	−41.94
	Arg (240)	2.5	–CD	Unfavorable bump	
**OsMTP11**	Glu (259) *	2.0	–CA –CO–NH	**Covalent bond** **Covalent bon**d	−48.60
	Lys (262)	2.4	–CD	Unfavorable bump	
OsMTP11.1	Phe (314)	2.4	–CO–NH	Unfavorable metal donor	−45.20
	Glu (315)	2.4	–C=O	Metal acceptor	
	His (317)	2.4	–C=O –CO–NH	Metal acceptor Unfavorable metal donor	
OsMTP12	Leu (225) *	2.0	–C=O	**Covalent bond**	−43.82
	Leu (226) *	2.3	–C	**Covalent bond**	
	Asn (228) *	1.6	–CO–NH	**Covalent bond**	
	Ser (229)	1.8	–CO–NH	Unfavorable bump	

* All covalent bands between Ga ions and specific AA residues are highlighted in bold font.

## Data Availability

The data is available at Supplementary Materials and the public databases mentioned in the study.

## Supplementary Materials

The following supporting information can be downloaded at: https://www.mdpi.com/article/10.3390/toxics12110831/s1, M1: Subcellular distribution of Ga. M2: RNA extraction and RT-qPCR analysis. Figure S1: The fraction (%) of Ga and mineral elements at different subcellular compartments of rice seedlings. NA refers to the amounts of Ga below the limit of Ga detection. Figure S2: Correlation analysis between all measured mineral elements and Ga concentrations in different subcellular compartments. Figure S3: The binding sites of individual rice MTPs with Ga^3+^ ions. Figure S4: Comparison of binding sites of OsMTP with Mn and Ga ions. Table S1: Interactome analysis of rice MTP genes. Table S2: The binding sites of Ga ions at specific motifs for individual rice MTP genes. Table S3: Member and number of CREs for individual rice MTP genes. Table S4: Interactome analysis of rice MTP genes.
