# Supplementary material for: The Role of Metal Tolerance Proteins (MTPs) Associated with the Homeostasis of Divalent Mineral Elements in Ga-Treated Rice Plants
Source: Toxics. 2024 Nov 20;12(11):831. doi: 10.3390/toxics12110831 (PMC11598383; doi:10.3390/toxics12110831)

M1: ***Subcellular distribution of Ga***

After harvesting, fresh plant materials was immediately weighed and homogenized in grinding mixture. The homogenate was kept at 4°C for further analysis. A known amount of root (0.5-0.8 g) and shoot (0.5-0.8 g) samples was taken for grinding. The grinding medium contains MES-Tris buffer (50 mM, pH 7.8), sucrose (0.25 mM), $MgCl_2$ (1 mM), and cysteine (10 mM) at 4°C. Briefly, the homogenate was centrifuged at 300 *g* for 30s. The pellet in the tube was designated as a cell wall fraction. The supernatant of the first centrifugation step was transferred into another tube using a pipette and then centrifuged at 20,000 *g* for 45 min. The supernatant of the second centrifugation step was collected as cytosol fraction and the pellet as organelle fraction. Finally, three subcellular fractions, i.e., cell wall, cytosol and organelles, were also digested using the digestion solution of 4:1 HNO_3-$HClO_4$ and measured by ICP-AES (PerkinElmer Optima 7000 DV). The instrument was set according to the subsequent conditions: a radio-frequency power of 1.3 kW, plasma gas flow rate of 13 L/min, and auxillary gas flow rate of 0.2 L/min. For the element measurement, Mg, Zn, Fe, Mn, Cu, and Ga at 285.213, 206.200, 231.617, 257.625, 327.409, and 417.206 nm were used, respectively. The detection limits for these elements were 0.23, 0.64, 0.49, 0.17, 0.06, and 0.06μg/L, respectively. Normally, we calibrated the instrument response and sample preparation variations by adding internal standards. After that, the standard curve was made using the serious of known concentrations of these compounds. During samples measuring process, we also checked the stability of the analytical process using the QC samples with known concentrations. The QC results were recorded and calculated the relative standard deviation (RSD). The RSD from the same QC sample, based on 3-4 times measurement is less than 10%, indicating that the experimental data is reliable.

M2: ***RNA extraction and RT-qPCR analysis***

RNA isolation from rice tissues i.e., roots and shoots of both treated and non-treated plants was done through an Ultrapure RNA Kit (CWBio, Taizhou, China). To remove any kind of genomic DNA contamination from the RNA extract, DNase I was used to get pure RNA. After washing the genomic DNA, an RNeasy MinElute Cleanup Kit (Qiagen, Hilden, Germany) was used to further purify the isolated RNA.

Ten genes from the rice MTP family were used for RT-qPCR after exposing to the three effective concentrations of Ga. Primer sequences used in the present study are listed in **Table S1**.

Each cycle of the RT-qPCR was set according to the subsequent conditions: 95°C for 10 s, 58°C for 30 s, and 72 °C for 32 s which was repeated 40 times. The RT-qPCR was performed by the 7500 Fast RT-qPCR system (Applied Biosystems, Life Technologies, Foster City, CA, USA) and SYBR green chemistry. *OsGAPDH* (LOC_Os08g03290.1) was used as a housekeeping gene (Yang et al., 2021). The relative expression of each targeted gene was calculated by the standard $2^{-\Delta\Delta CT}$ method (Schmittgen and Livak 2008).

Table S1：Primer sequences of rice MTP genes used

Gene name	Locus identifier	Primer sequences(5'-3')	Amplicon size (bp)
OsMTP1	LOC_Os05g03780.1	F-GCCACCAGCCTTGAGAATG<br>R-GTCTGCGTCCTGAGTGATTG	129
OsMTP5	LOC_Os02g58580.1	F-GTTTCTTTCATTGGGGGT<br>R-GGTTTGGATGGTCAGGTC	346

OsMTP6	LOC_Os03g22550.1	F-TATCTGTCAAAGAAGGGC<br>R-TGATTGGTTGTAGAAGCG	282
OsMTP7	LOC_Os04g23180.1	F-AGCAGCAGAAGGAATGAG<br>R-TGAACAGCCACCAAAGAT	132
OsMTP8	LOC_Os02g53490.1	F-CATCCACGCTTGATTCCT<br>R-TCTCTCCCGCCTTGTTCT	216
OsMTP8.1	LOC_Os03g12530.1	F-GAGCAAAAGCAGAGTGAG<br>R-CGAGAGATGTGTGAACCA	174
OsMTP9	LOC_Os01g03914.1	F-GCCAGTGGGCATAATAGT<br>R-TGAGTCCCGAAGGTGTAG	416
OsMTP11	LOC_Os01g62070.1	F-CTCAACTTCGACGGCTTCC<br>R-CAACCTTCTCACGCTCTTCC	223
OsMTP11.1	LOC_Os05g38670.1	F-CCAATCGGCTTCACCAGAG<br>R-GCAGAACAATATCCACCTCAAC	137
OsMTP12	LOC_Os08g32650.1	F-TGTTGTTGTGGATCGCCAGT<br>R-TCGACTTCCGTTCGTTCAGG	132
OsGAPDH1	LOC_Os08g03290.1	GACAGCAGGTCGAGCATCTTC<br>CAGGCGACAAGCTTGACAAAG	74

The detailed information of TMDs highlighted in different colors located at the sequences of individual rice MTPs

OsMTP1: TMDI; TMDII; TMDIII; TMDIV; TMDV; TMDVI

MDSHNSAPPQIAEVRMDISSSTSVAAGNKVCRGAACDFSDSSNSSKDARERMASMRKLIIAVILCIIF
MAVEVVGGIKANSLAILTDAAHLLSDVAAFAISLFSLWAAGWEATPQQSYGFFRIEILGALVSIQLIW
LLAGILVYEAIVRLINESGEVQGSLMFAVSAFGLFVNIIMAVLLGHDHGHGHGHGHGHSHDHDH
GGSDHDHHHHEDQEHGHVHHHEDGHGNSITVNLHHHPGTGHHHHDAEEPLLKSDAGCDSTQSG
AKDAKKARRNINVHSAYLHVLGDSIQSIGVMIGGAIIWYKPEWKIIDLICTLIFSVIVLFTTIKMLRNI
LEVLMESTPREIDATSLENGLRDMDGVVAVHELHIWAITVGKVLLACHVTITQDADADQMLDKVI
GYIKSEYNISHVTIQIERE

MTP5: TMDI; TMDII; TMDIII; TMDIV

MLSISPLVVESSLSPCLPWLPPEPNLITSTPTGASLPQFTPLFFNLPLPLIHCCLYSAIKDWRSLLPLQM
PQLFLLFLSFSLAVEALHAFMQDESEHKHYLIVSAVTNLLVNLLGVWFFRSYARVNIVYRKAEDMN
YHSVCLHVLADSVRSAGLILASWFLSLGVENAEVLCLGIVSVAVFMLVLPLFKATGNILLQIAPGNV
PPSALTKCFRQITACEDVSEVCQGRFWELVPGHAVGSLDIRVKNGGDCQSVLDYVHGLYQDLGIQD
LTIQTDE

MTP6: TMDI; TMDII; TMDIII; TMDIV; TMDV; TMDVI

MGFRLAHLAACVARAAASSSRLRGPRPAASALVAPLLASPWEPSGGGQPHWLVPSRGHVGHSHHH
HHGEEVGGEASERIFRLGLAADVVLTVGKAVTGYLSGSTAIAADAAHSLSDIVLSGVALLSYKAAK
APRDKEHPYGHGKFESLGALGISSMLLVTAGGIAWHAFDVLQGVMSSAPDIIGNVSHAHHSHGSSG
HHHGIDLEHPILALSVTAFAISVKEGLYWITKRAGEKEGSGLMKANAWHHRSDAISSVVALLGVGG

SILGVPYLDPLAGLVVSGMILKAGVHTGYESVLELVDAAVDPSLLQPIKETILQVDGVKGCHRLRG
RKAGTSLYLDVHIEVYPFLSVSAAHDIGETVRHQIQKSHNQVAEVFIHIGSLQPLNQNAL

MTP7: TMDI; TMDII; TMDIII; TMDIV; TMDV
MRRPFAAAAALRLRLLSSSSSSSSSSSSLPRLPSSPYPLHHLLLLSRRSGDHHDHDHPSPPPPFSPRPL
LASGVLGLSRWRARARALPPAPSPPRGPVADAPPVRLTLSRSYSLRVAKAKKKAHFDDEHSHRAVN
TALWCNFLVFSLKFGVWLSTSSHVMLAELVHSVADFANQALLAYGLSSSRRAPDALHPYGYSKER
FVWSLISAVGIFCLGSGATIVHGVQNLWNSQPPENIHYAALVIGGSFLIEGASLLVAIKAVRKGAAAE
GMSIRDYIWRGHDPTSVAVMTEDGAAVTGLAIAGASLVAVQTTGNAMYDPIGSIIVGNLLGMVAIFL
IQRNRHALIGRAIDDHDMQRVLEFLKADPVVDALYDCKSEVIGPGFFRFKAEIDFNGVVLVQNYLE
RTGRGEWAKQFREASLSKDDTELIRVMSNYGEDVVEALGYEVDRLESEIQKIVPGIKHVDIEAHNP
EGLSL

MTP8: TMDI; TMDII; TMDIII; TMDIV; TMDV; TMDVI
MDGDDRRTPLLGGEGGSTRPPSLRRRDSARSLRSTFLSRLPDKVRGGGDPERPAADVDLTRAKGLS
QGEKEYYEKQLATLKIFEEVEALCMPGEFESDAEVLELEDKEQKQSESAMKISNYANIILLVFKVYA
TIKTGSMAIAASTLDSLLDFLAGGILYFTHLTMKSVNIYKYPIGKLRVQPVGIIVFAAIMATLGFQVLI
QAIEQLVENKAGEKMTPEQLIWLYSIMLSATVVKLALYIYCRSSGNSIVQAYAKDHYFDVVTNVVG
LVAAVLGDKFFWWIDPVGAVLLAVYTIVNWSGTVYENAVTLVGQCAPSDMLQKLTYLAMKHDPR
VRRVDTVRAYSFGALYFVEVDIELSEDMRLGEAHSIGESLQDKIEKLPEVERAFVHVDFESTHKPEH
RVRSRLPSTEP

MTP8.1: TMDI; TMDII; TMDIII; TMDIV; TMDV; TMDVI
MEAKGENDARAPLLAERRRNSVGSMRGEFVSRLPKKVLDAVDPERPSHVDFSRSKGLREGEKEY
YEKQFATLRSFEEVDSIEESNVMSEEDDIAEQKQSEFAMKISNYANMILLALKIYATIKSGSIAIAAST
LDSLLDLMAGGILWFTHLSMKSINVYKYPIGKLRVQPVGIIIFAAVMATLGFQVFVQAVEKLIVNET
PDKLTPVQLTWLYSIMIFATVVKLALWLYCRTSGNKIVRAYAKDHYFDVVTNVVGLAAAVLGDMF
YWWIDPVGAIALAVYTITNWSGTVWENAVSLVGESAPPEMLQKLTYLAIRHHPQIKRVDTVRAYTF
GVLYFVEVDIELPEELPLKEAHAIGESLQIKIEELPEVERAFVHLDFECDHKPEHNILSKLPSSQP

MTP9: TMDI; TMDII; TMDIII; TMDIV; TMDV; TMDVI
MGSRGRRGGGERETETEEDETWKLRVGDDFTVPERFHRKPPFFSRIFPAGSHGKHRKIAKYYKKQE
NLLKDFSEMETMNEIGSLDQNAPTEEELRQMAKGERLAINLSNIINLILFIGKVLASVESLSMAVIAS
TLDSLLDLLSGFILWFTAHAMKKPNKYSYPIGKRRMQPVGIIVFASVMGTLGFQVLIESGRQLITNE
HQVFDHRKELWMIGSMSSVAVVKFFLMLYCRSFKNEIVRAYAQDHFFDVITNSVGLVSALLAVRYK
WWMDPVGAILIAVYTITTWARTVVENVGTLIGRSAPAEYLTKLTYLIWNHHEEIRHIDTVRAYTFGT
HYFVEVDIVLPGDMPLSHAHDIGESLQEKLEQLPEVERAFVHVDFEFTHRPEHKAEV

MTP11: TMDI; TMDII; TMDIII; TMDIV; TMDV; TMDVI
MAAAVAGGGEEGEELLLLSAVEAGSFGGGGDGGGAGAAAEKSWRLNFDGFRPPEVQQERRPPRG
LHHHCLGVLSQGPEDVVAEYYQQQVEMLEGFNEMDTLTDRGFLPGMSKEEREKVARSETLAIRLS
NIANMVLFAAKVYASVRSGSLAIIASTLDSLLDLLSGFILWFTAFSMQTPNPYRYPIGKKRMQPLGIL
VFASVMATLGLQIILESVRSLLSDGDEFSLTKEQEKWVVDIMLAVTLVKLALVLYCRTFTNEIVKAY
AQDHFFDVITNMIGLVAALLATYIEGWIDPVGAIILAIYTIRTWSMTVLENVHSLVGQSASPEYLQKL

TYLCWNHHKAVRHIDTVRAYTFGSHYFVEVDIVLPSSMPLQEAHDIGEALQEKLERLPEIERAFVH
LDYEFTHRPEHALSHEK

MTP11.1: TMDI; TMDII; TMDIII; TMDIV

MAAAAGVAAGTGRGSGEGEELLPNAVEGDGGCGGGGTCAGDRPWRLNFDGLRRPEAHQEKPPR
RFHDRLGGLVQSPGDDVAEYYQQQSELLEGFNEMDTLTDRGFLPGMSKEECEKVARSEALAIRLSN
IANMVLFAAKVYASIRSGSLAIIASTLDSLLDLLSGFILWFTAFSKKTSNPYRYPIGKRRMQPLGILVF
ASVMATLGLQIILESTRSLFYDGDTFRLTKEQEKWVVDIMLSVTSVKLLLVVYCRSFTNEILAIYTIR
TWSMTVLENVHSLVGQSASPEYLQKLTYLCWNHHKAVRHIDTVRAYTFGSHYFVEVDIVLPCDMP
LQEAHDIGEAPQEKLESLPEIERAFVHLDYEFTHQPEHARSHDTL

MTP12: TMDI; TMDII; TMDIII; TMDIV; TMDV; TMDVI

MGPVRHILNERKSRKIAAFLLINTAYMFVEFTSGFMSDSLGLISDACHMLFDCAALAIGLYASYIAR
LPANGLYNYGRGRFEVLSGYVNAVFLVLVGALIVLESFERILEPREISTSSLLTVSIGGLVVNVIGLVF
FHEEHHHAHGEAHSCNGGLQSSENHNKSRNRHHIDHNMEGIFLHVLADTMGSVGVVISTLLIKYK
GWLIADPICSVFISIMIVSSVLPLLRNSAEILLQRVPRSLEKDIKEALDDVMKIKGVIGVHNFHVWNL
TNTDIVGTFHLHITTEADKSSIREKASDIFHEAGIQDLTIQIECVKR

Table S2: The binding sites of Ga ions at specific motifs for individual rice MTP genes

Gene names	Specific motifs at the TMD regions for individual MTPs	Binding sites at the motifs
OsMTP1	10, 13, 32, 54; 12, 16, 34, 39	Motif 1
OsMTP5	12, 24, 26, 30, 33, 40, 62	NA
OsMTP12	10, 12, 13, 16, 21, 21, 32, 33, 34, 44, 51, 54, 63	NA
OsMTP6	10, 23, 35, 37, 38, 46, 50, 59, 60, 67	NA
OsMTP7	18, 21, 26, 29, 35, 40, 41, 47, 57, 50, 62, 69	NA
OsMTP8	2, 4, 5, 7, 11	Motif 4
OsMTP8.1	2, 4, 5, 7, 11	Motif 4
OsMTP9	2, 4, 5, 11, 47	Motif 4
OsMTP11	2, 4, 5, 7, 11	Motif 4
OsMTP11.1	2, 5, 7, 11, 68	NA

Table S3: Member and number of CREs for individual rice MTP genes

Gene	Light responsive	Phytohormone responsive	Environmental stress responsive	General regulatory elements	Regulation of plant development	Binding responsive	Total
OsMTP1	8	4	3	3	2	0	20
OsMTP5	6	2	4	2	0	0	14
OsMTP6	3	4	3	3	0	1	14
OsMTP7	4	6	2	3	1	1	17
OsMTP8	4	2	4	2	0	1	13
OsMTP8.1	7	4	3	3	0	1	18
OsMTP9	7	3	5	3	2	1	21
OsMTP11	5	5	3	3	2	1	19
OsMTP11.1	8	6	3	3	0	0	20

OsMTP12	2	3	3	3	1	0	12

Fig. S1: The fraction (%) of Ga and mineral elements at different subcellular compartments of rice seedlings. ND refers to the amounts of Ga below the limit of Ga detection.

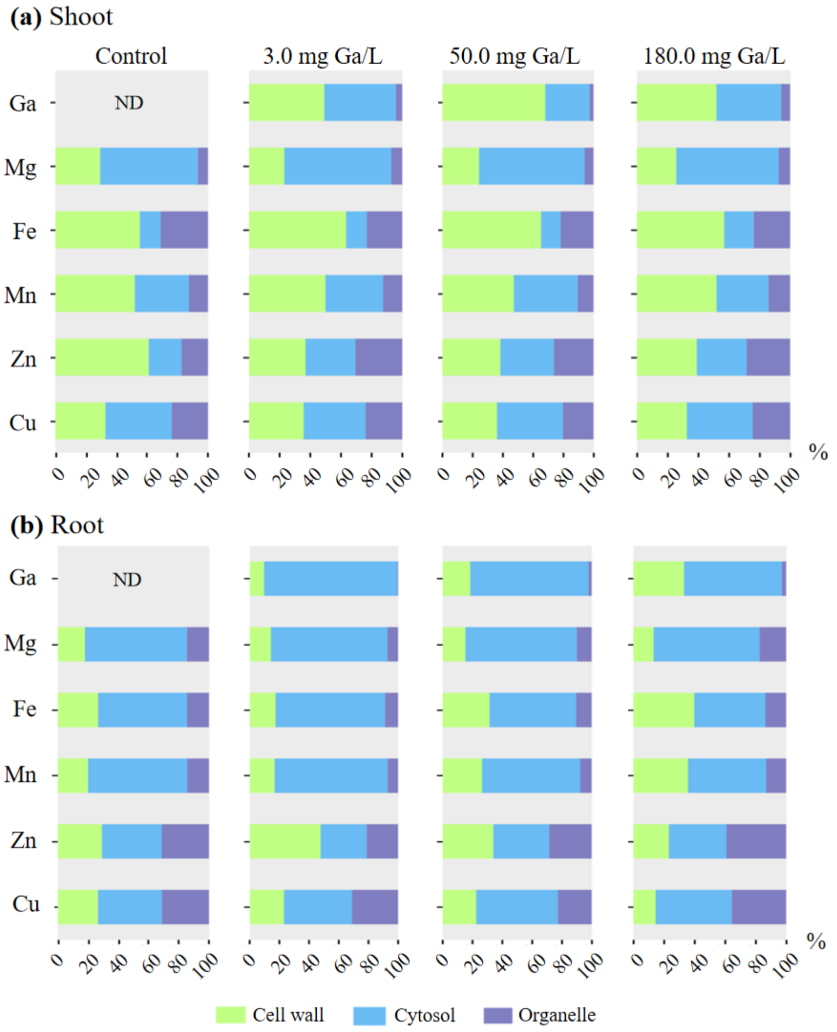

Fig. S2: Correlation analysis between all measured mineral elements and Ga concentrations in different subcellar compartments

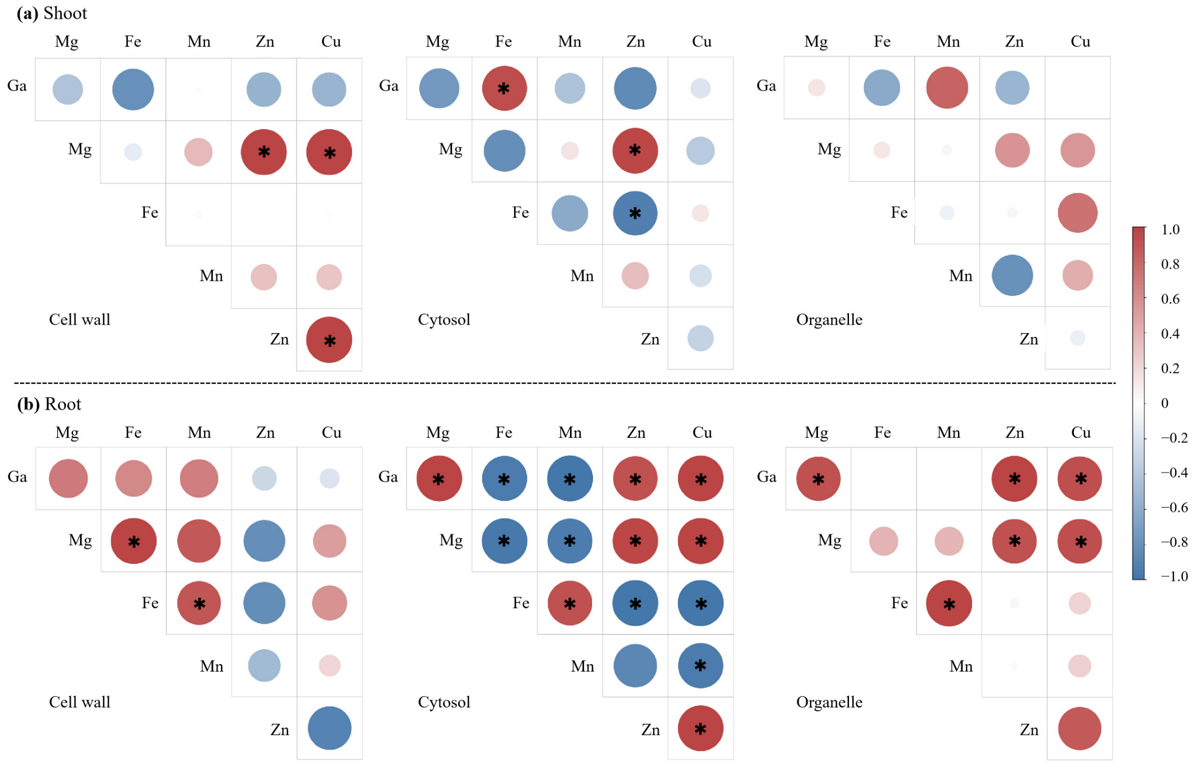

Fig. S3: The binding sites of individual rice MTPs with Ga^{3+} ions

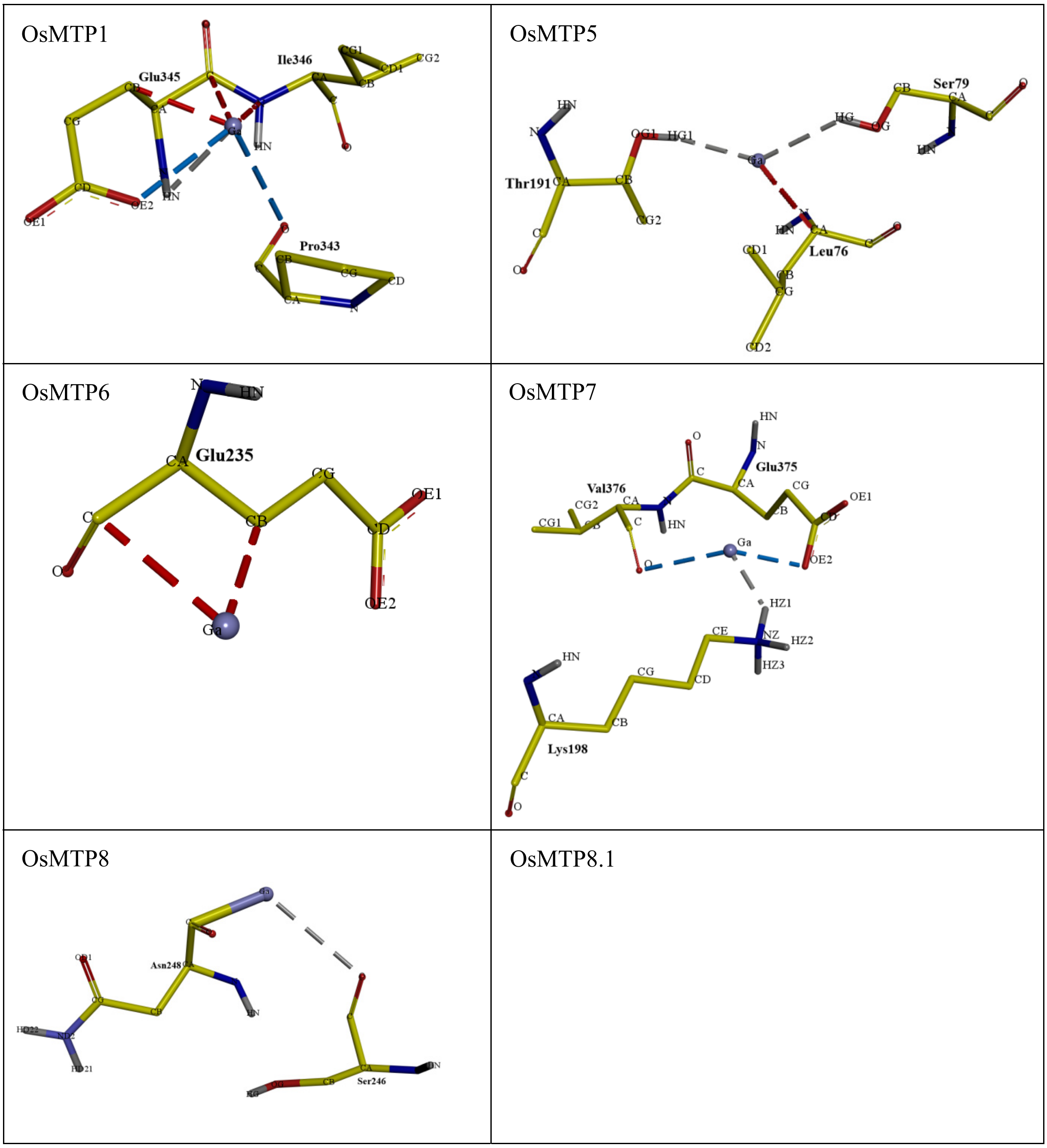

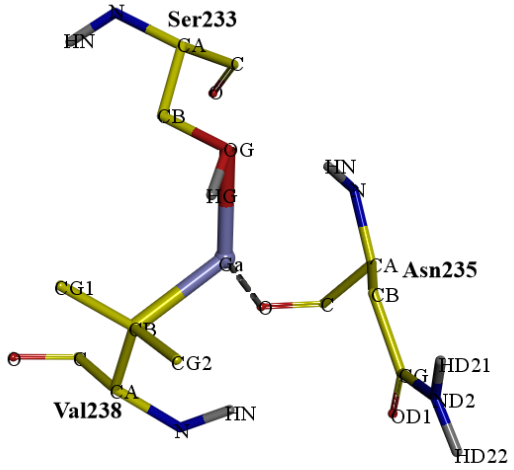

OsMTP9

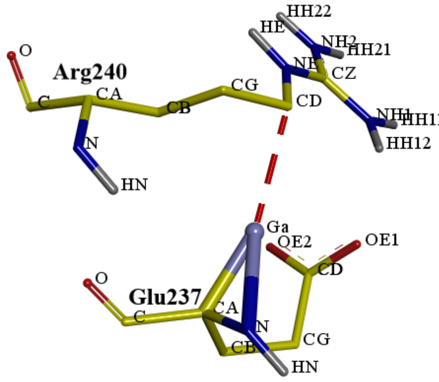

OsMTP11

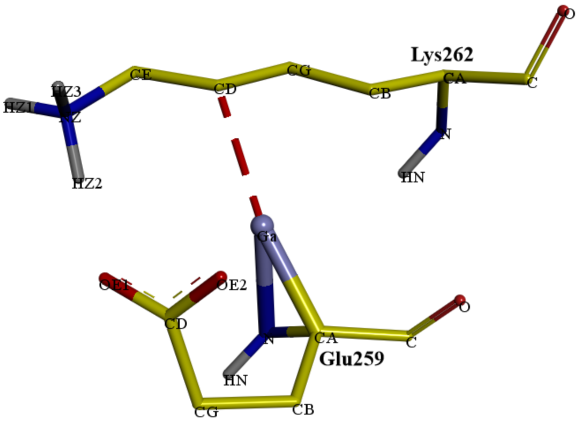

OsMTP11.1

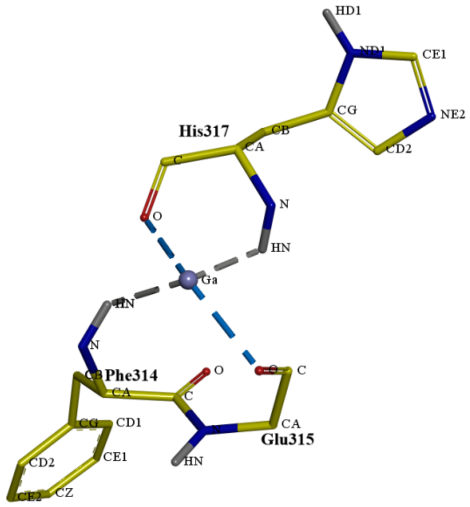

OsMTP12

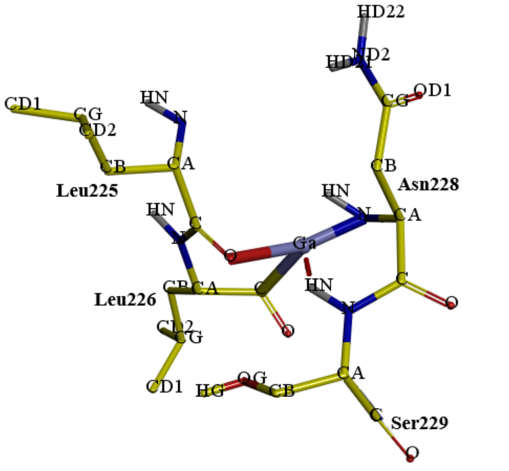

Table S4: Interactome analysis of rice MTP genes

Rice MTP gene	Interactive partner gene	Function description
OsMTP1	*OsFPN1*	Ferroportin, Mediation of cobalt and nickel transport, Detoxification of cobalt and nickel

	OsHMA1	Similar to Potential cadmium/zinc-transporting ATPase HMA1
	OsHMA2	P-Type heavy metal ATPase, Delivery of zinc to developing tissues
	OsHMA3	Heavy metal-transporting P_1B-ATPase, Root-to-shoot cadmium translocation
	OsHMA4	Heavy metal P_1B-type ATPase, Copper-transporting ATPase, Control of copper accumulation in rice grain
	OsHMA5	Heavy metal P-type ATPase, Xylem loading of copper
	OsHMA6	Heavy metal P-Type ATPase 6, Copper efflux protein
	OsHMA7	Heavy metal transporter protein, Regulation of yield and grain iron-zinc content
	OsHMA8	Copper-translocating P-type ATPase family protein
	OsHMA9	P-type heavy-metal ATPase, Metal efflux transport
	OsIRT1	Fe^{2+} transporter, Uptake of iron
	OsIRT2	Fe^{2+} transporter, Uptake of iron
	OsNramp1	Integral membrane protein, Metal ion transport
	OsNramp3	Integral membrane protein, Metal ion transport
	OsSOD-Cu/Zn	Superoxide dismutase, copper/zinc binding domain containing protein
	OsSOD-Fe1	Similar to Superoxide dismutase [Fe], chloroplast
	OsSOD-Fe2	Splicing variant of the iron-superoxide dismutase
	OsSOD-Mn	Manganese superoxide dismutase, Heat-stress tolerance
	OsVIT1;2	Vacuolar membrane transporter, iron and zinc translocation between flag leaves and seeds
	OsVIT2	Vacuolar membrane transporter, iron and zinc translocation between flag leaves and seeds
	OsVTL5	Vacuolar iron transporter homologue, Iron homeostasis
	OsZIP1	Zinc transporter, Metal-detoxified transporter, Detoxification of excess zinc, copper and cadmium
	OsZIP11	ZRT/IRT-like protein family metal transporter, Mediation of iron accumulation for rice growth and development

	OsZIP14	Zinc/iron permease family protein
	OsZIP16	ZRT/IRT-like protein, Metal transporter, Growth and development under cadmium stress
	OsZIP3	Zinc transporter, Preferential distribution of zinc to developing tissues
	OsZIP6	Transition metal ion transporter, Ion transport
	OsZIP7	Plasma membrane cadmium transporter of the zinc-regulated
	OsZIP8	Zinc transporter, zinc uptake and distribution
	OsZN	Nickel/cobalt transporter
	OsZNL	Zebra-necrosis like protein, Nickel/cobalt transporter, high-affinity domain containing protein
OsMTP5	*OsHMA1*	Similar to Potential cadmium/zinc-transporting ATPase HMA1
	OsHMA2	P-Type heavy metal ATPase, Delivery of Zinc to developing tissues
	OsHMA3	Heavy metal-transporting P1B-ATPase, Root-to-shoot cadmium translocation
	OsHMA4	Heavy metal P_1B-type ATPase, copper-transporting ATPase, Control of copper accumulation in rice grain
	OsHMA5	Heavy metal P-type ATPase, Xylem loading of copper
	OsHMA6	Heavy metal P-Type ATPase 6, Copper efflux protein
	OsHMA7	Heavy metal transporter protein, Regulation of yield and grain iron-Zinc content
	OsHMA8	Copper-translocating P-type ATPase family protein
	OsHMA9	P-type heavy-metal ATPase, Metal efflux transport
	OsSOD-Cu/Zn	Superoxide dismutase, copper/zinc binding domain containing protein
	OsSOD-Fe1	Similar to Superoxide dismutase [Fe], chloroplast
	OsSOD-Fe2	Splicing variant of the iron-superoxide dismutase
	OsSOD-Mn	Manganese superoxide dismutase, Heat-stress tolerance
	OsZIP11	ZRT/IRT-like protein family metal transporter, Mediation of iron accumulation for rice growth and development

	OsZIP13	Zinc/iron permease family protein
	OsZIP14	Zinc/iron permease family protein
	OsZIP16	ZRT/IRT-like protein, Metal transporter, Growth and development under cadmium stress
	OsZN	Nickel/cobalt transporter
	OsZNL	Zebra-necrosis like protein, Nickel/cobalt transporter, high-affinity domain containing protein
OsMTP6	*OscMDH*	NAD-dependent cytosolic malate dehydrogenase
	OsHMA9	P-type heavy-metal ATPase, Metal efflux transport
	OsMDH1	Malate dehydrogenase, Salt stress tolerance
	OsMDH2.1	Malate dehydrogenase 2.1
	OsMDH3.1	Malate dehydrogenase 3.1
	OsMDH4.1	Malate dehydrogenase 4.1
	OsMDH5.1	Malate dehydrogenase 5.1
	OsMDH6.1	Malate dehydrogenase 6.1, Positive regulation of salt tolerance
	OsMDH7.1	Malate dehydrogenase 7.1
	OsMDH8.1	Malate dehydrogenase, Salt tolerance
	OsMDH8.2	Malate dehydrogenase 8.2
	OsMDH12.1	Malate dehydrogenase, Salt stress response
	OsmMDH	Mitochondrial malate dehydrogenase, Flowering and seeding
	OsSOD-Cu/Zn	Superoxide dismutase, copper/zinc binding domain containing protein
OsMTP7	*OscMDH*	NAD-dependent cytosolic malate dehydrogenase
	OsMDH1	Malate dehydrogenase, Salt stress tolerance
	OsMDH2.1	Malate dehydrogenase 2.1

	OsMDH3.1	Malate dehydrogenase 3.1
	OsMDH4.1	Malate dehydrogenase 4.1
	OsMDH5.1	Malate dehydrogenase 5.1
	OsMDH6.1	Malate dehydrogenase 6.1, Positive regulation of salt tolerance
	OsMDH7.1	Malate dehydrogenase 7.1
	OsMDH8.1	Malate dehydrogenase, Salt tolerance
	OsMDH8.2	Malate dehydrogenase 8.2
	OsMDH12.1	Malate dehydrogenase, Salt stress response
	OsmMDH	Mitochondrial malate dehydrogenase, Flowering and seeding
	OsSOD-Cu/Zn	Superoxide dismutase, copper/zinc binding domain containing protein
	OsZIP11	ZRT/IRT-like protein family metal transporter, Mediation of iron accumulation for rice growth and development
	OsZIP13	Zinc/iron permease family protein
	OsZIP14	Zinc/iron permease family protein
	OsZIP16	ZRT/IRT-like protein, Metal transporter, Growth and development under Cd stress
OsMTP8	*OscMDH*	NAD-dependent cytosolic malate dehydrogenase
	OsFPN1	Ferroportin, Mediation of cobalt and nickel transport, Detoxification of cobalt and nickel
	OsMDH1	Malate dehydrogenase, Salt stress tolerance
	OsMDH2.1	Malate dehydrogenase 2.1
	OsMDH3.1	Malate dehydrogenase 3.1
	OsMDH4.1	Malate dehydrogenase 4.1
	OsMDH5.1	Malate dehydrogenase 5.1
	OsMDH6.1	Malate dehydrogenase 6.1, Positive regulation of salt tolerance

	OsMDH7.1	Malate dehydrogenase 7.1
	OsMDH8.1	Malate dehydrogenase, Salt tolerance
	OsMDH8.2	Malate dehydrogenase 8.2
	OsMDH12.1	Malate dehydrogenase, Salt stress response
	OsmMDH	Mitochondrial malate dehydrogenase, Flowering and seeding
	OsSOD-Cu/Zn	Superoxide dismutase, copper/zinc binding domain containing protein
OsMTP8.1	*OscMDH*	NAD-dependent cytosolic malate dehydrogenase
	OsFPN1	Ferroportin, Mediation of cobalt and nickel transport, Detoxification of cobalt and nickel
	OsMDH1	Malate dehydrogenase, Salt stress tolerance
	OsMDH2.1	Malate dehydrogenase 2.1
	OsMDH3.1	Malate dehydrogenase 3.1
	OsMDH4.1	Malate dehydrogenase 4.1
	OsMDH5.1	Malate dehydrogenase 5.1
	OsMDH6.1	Malate dehydrogenase 6.1, Positive regulation of salt tolerance
	OsMDH7.1	Malate dehydrogenase 7.1
	OsMDH8.1	Malate dehydrogenase, Salt tolerance
	OsMDH8.2	Malate dehydrogenase 8.2
	OsMDH12.1	Malate dehydrogenase, Salt stress response
	OsmMDH	Mitochondrial malate dehydrogenase, Flowering and seeding
	OsSOD-Cu/Zn	Superoxide dismutase, copper/zinc binding domain containing protein
	OsVIT1;2	Vacuolar membrane transporter, iron and zinc translocation between flag leaves and seeds
	OsVIT2	Vacuolar membrane transporter, iron and zinc translocation between flag leaves and seeds

OsMTP9	*OscMDH*	NAD-dependent cytosolic malate dehydrogenase
	OsMDH1	Malate dehydrogenase, Salt stress tolerance
	OsMDH2.1	Malate dehydrogenase 2.1
	OsMDH3.1	Malate dehydrogenase 3.1
	OsMDH4.1	Malate dehydrogenase 4.1
	OsMDH5.1	Malate dehydrogenase 5.1
	OsMDH6.1	Malate dehydrogenase 6.1, Positive regulation of salt tolerance
	OsMDH7.1	Malate dehydrogenase 7.1
	OsMDH8.1	Malate dehydrogenase, Salt tolerance
	OsMDH8.2	Malate dehydrogenase 8.2
	OsMDH12.1	Malate dehydrogenase, Salt stress response
	OsmMDH	Mitochondrial malate dehydrogenase, Flowering and seeding
	OsSOD-Cu/Zn	Superoxide dismutase, copper/zinc binding domain containing protein
OsMTP11	*OscMDH*	NAD-dependent cytosolic malate dehydrogenase
	OsMDH1	Malate dehydrogenase, Salt stress tolerance
	OsMDH2.1	Malate dehydrogenase 2.1
	OsMDH3.1	Malate dehydrogenase 3.1
	OsMDH4.1	Malate dehydrogenase 4.1
	OsMDH5.1	Malate dehydrogenase 5.1
	OsMDH6.1	Malate dehydrogenase 6.1, Positive regulation of salt tolerance
	OsMDH7.1	Malate dehydrogenase 7.1
	OsMDH8.1	Malate dehydrogenase, Salt tolerance

	OsMDH8.2	Malate dehydrogenase 8.2
	OsMDH12.1	Malate dehydrogenase, Salt stress response
	OsmMDH	Mitochondrial malate dehydrogenase, Flowering and seeding
	OsSOD-Cu/Zn	Superoxide dismutase, copper/zinc binding domain containing protein
OsMTP11.1	*OscMDH*	NAD-dependent cytosolic malate dehydrogenase
	OsMDH1	Malate dehydrogenase, Salt stress tolerance
	OsMDH2.1	Malate dehydrogenase 2.1
	OsMDH3.1	Malate dehydrogenase 3.1
	OsMDH4.1	Malate dehydrogenase 4.1
	OsMDH5.1	Malate dehydrogenase 5.1
	OsMDH6.1	Malate dehydrogenase 6.1, Positive regulation of salt tolerance
	OsMDH7.1	Malate dehydrogenase 7.1
	OsMDH8.1	Malate dehydrogenase, Salt tolerance
	OsMDH8.2	Malate dehydrogenase 8.2
	OsMDH12.1	Malate dehydrogenase, Salt stress response
	OsmMDH	Mitochondrial malate dehydrogenase, Flowering and seeding
	OsSOD-Cu/Zn	Superoxide dismutase, copper/zinc binding domain containing protein
	OsVIT1;2	Vacuolar membrane transporter, Fe and Zn translocation between flag leaves and seeds
OsMTP12	*OsHMA1*	Similar to Potential cadmium/zinc-transporting ATPase HMA1
	OsHMA2	P-Type heavy metal ATPase, Delivery of zinc to developing tissues
	OsHMA3	Heavy metal-transporting P_1B-ATPase, Root-to-shoot cadmium translocation
	OsHMA4	Heavy metal P_1B-type ATPase, Cu-transporting ATPase, Control of copper accumulation in rice grain

	OsHMA5	Heavy metal P-type ATPase, Xylem loading of copper
	OsHMA6	Heavy metal P-Type ATPase 6, Copper efflux protein
	OsHMA7	Heavy metal transporter protein, Regulation of yield and grain iron-zinc content
	OsHMA8	Copper-translocating P-type ATPase family protein
	OsHMA9	P-type heavy-metal ATPase, Metal efflux transport
	OsIRT1	Fe^{2+} transporter, Uptake of iron
	OsNramp3	Integral membrane protein, Metal ion transport
	OsSOD-Cu/Zn	Superoxide dismutase, copper/zinc binding domain containing protein
	OsSOD-Fe1	Similar to superoxide dismutase [Fe], chloroplast
	OsSOD-Fe2	Splicing variant of the iron-superoxide dismutase
	OsSOD-Mn	Manganese superoxide dismutase, Heat-stress tolerance
	OsVIT1;2	Vacuolar membrane transporter, iron and zinc translocation between flag leaves and seeds
	OsVIT2	Vacuolar membrane transporter, iron and zinc translocation between flag leaves and seeds
	OsZIP11	ZRT/IRT-like protein family metal transporter, Mediation of iron accumulation for rice growth and development
	OsZIP13	Zinc/iron permease family protein
	OsZIP14	Zinc/iron permease family protein
	OsZIP16	ZRT/IRT-like protein, Metal transporter, Growth and development under cadmium stress
	OsZN	Nickel/cobalt transporter
	OsZNL	Zebra-necrosis like protein, Nickel/cobalt transporter, high-affinity domain containing protein

Fig. S4: Comparison of binding sites of OsMTP with Mn and Ga ions

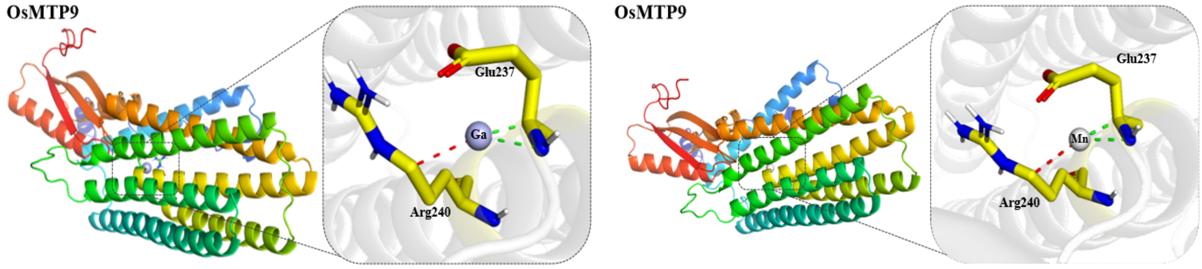

Supplement: Supplementary file 1 [file toxics-12-00831-s001.zip › toxics-3314580-supplementary.pdf]
